# Supplementary material for: The protein disulfide isomerase P4HB/PDIA1 modulates cellular and misfolded forms of the prion protein
Source: PLoS Pathog. 2026 Aug 18;22(8):e1014536. doi: 10.1371/journal.ppat.1014536 (PMC13515023; doi:10.1371/journal.ppat.1014536)

The Protein Disulfide Isomerase P4HB/PDIA1 Modulates Cellular and Misfolded Forms of the Prion Protein

Genki Amano[1], Hamza Arshad[1,2], Zeel Patel[1], Gerold Schmitt-Ulms[1,3], and Joel C. Watts[1,2, §]

[1]Tanz Centre for Research in Neurodegenerative Diseases, University of Toronto, Toronto, Ontario, Canada, M5T 0S8

[2]Department of Biochemistry, Temerty Faculty of Medicine, University of Toronto, Toronto, Ontario, Canada, M5S 1A8

[3]Department of Laboratory Medicine and Pathobiology, Temerty Faculty of Medicine, University of Toronto, Toronto, Ontario, Canada, M5S 1A8

Uncropped blots

Fig 1

B

IB: Actin

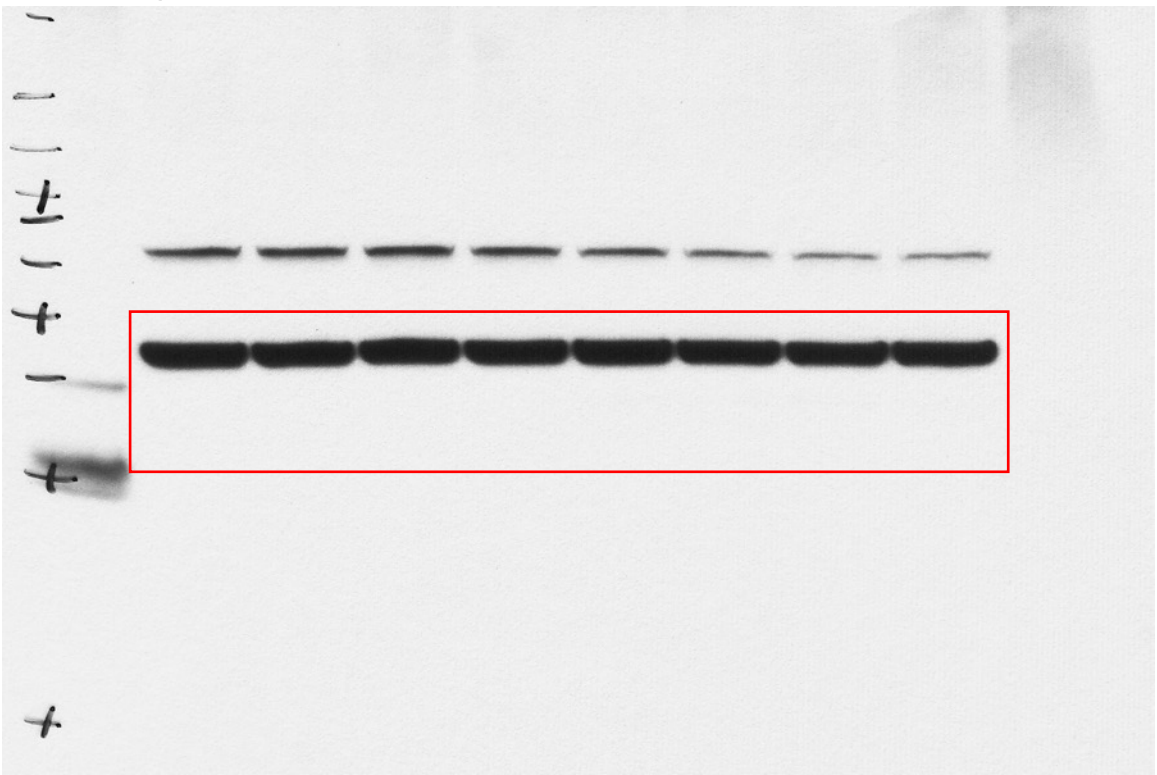

IB: PrP (D18)

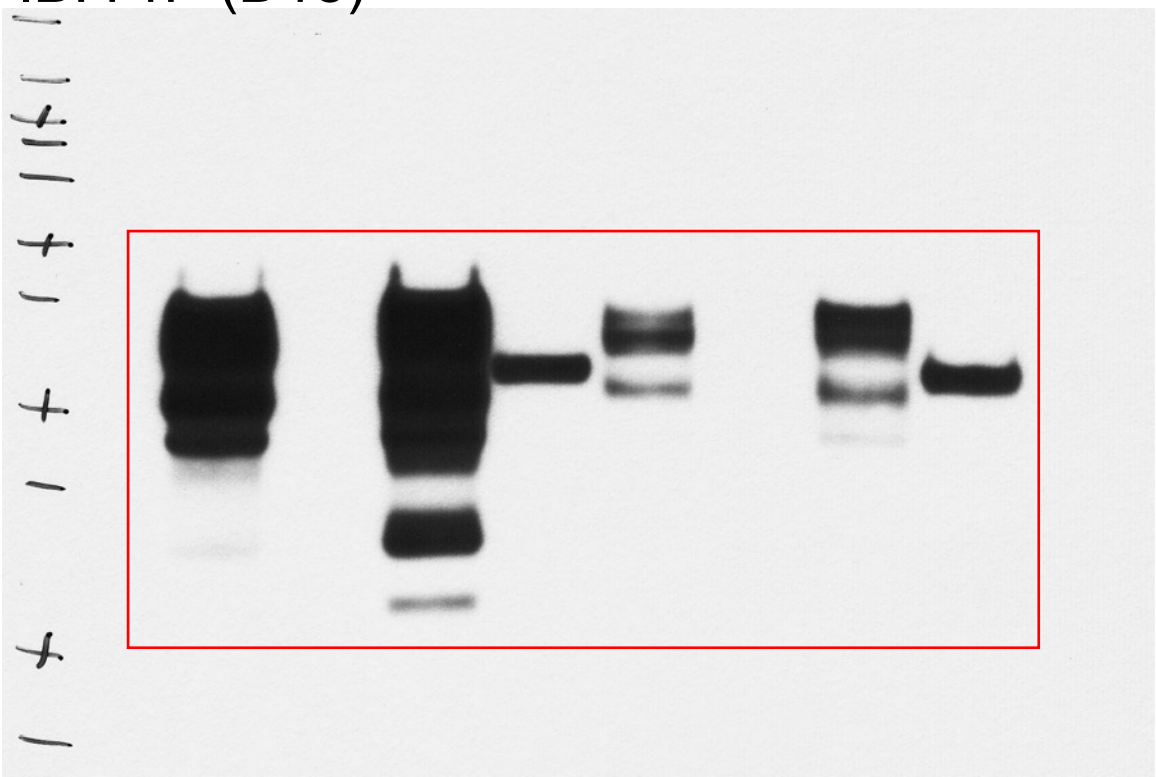

E

IB: Actin

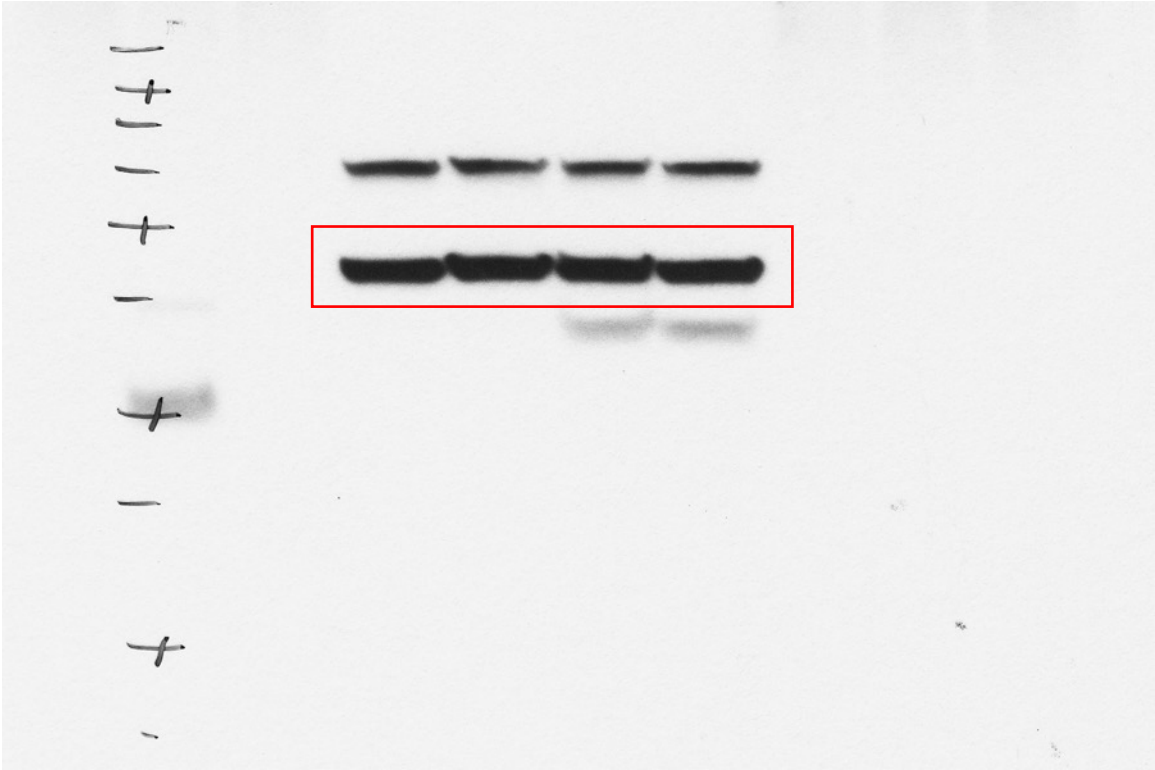

IB: PrP(D18)

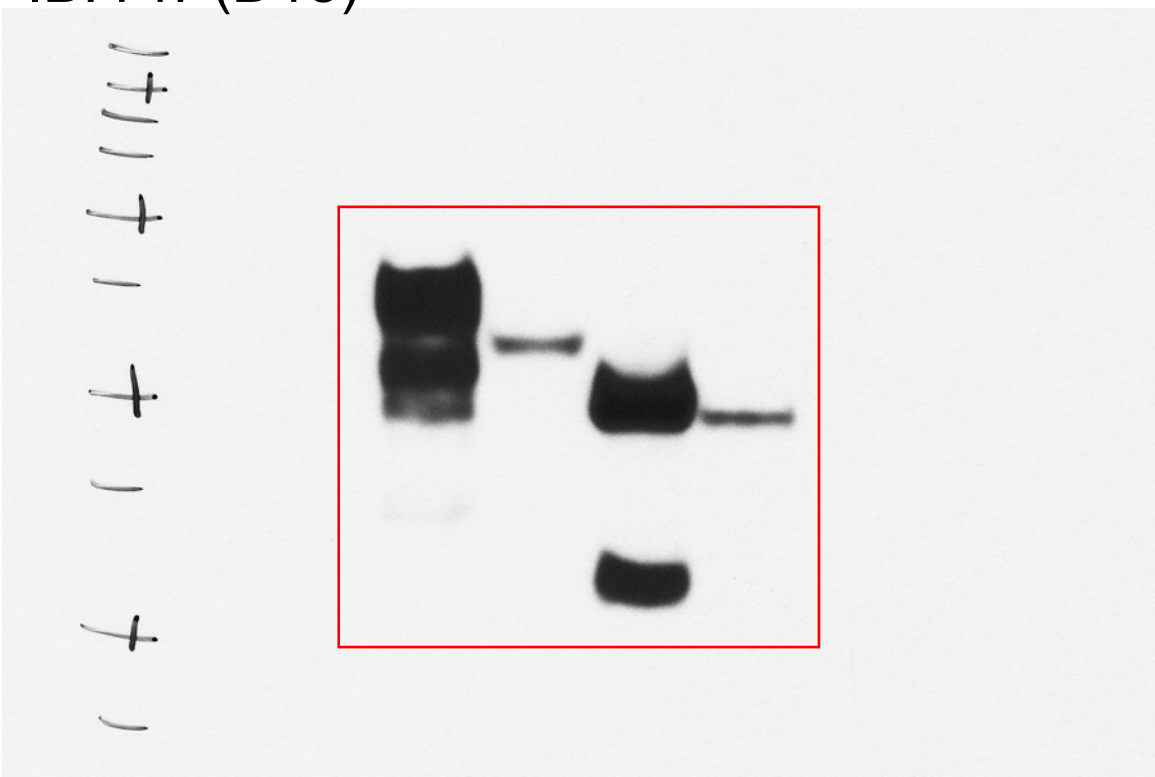

Fig 2

B

IB: PrP (SAF32)

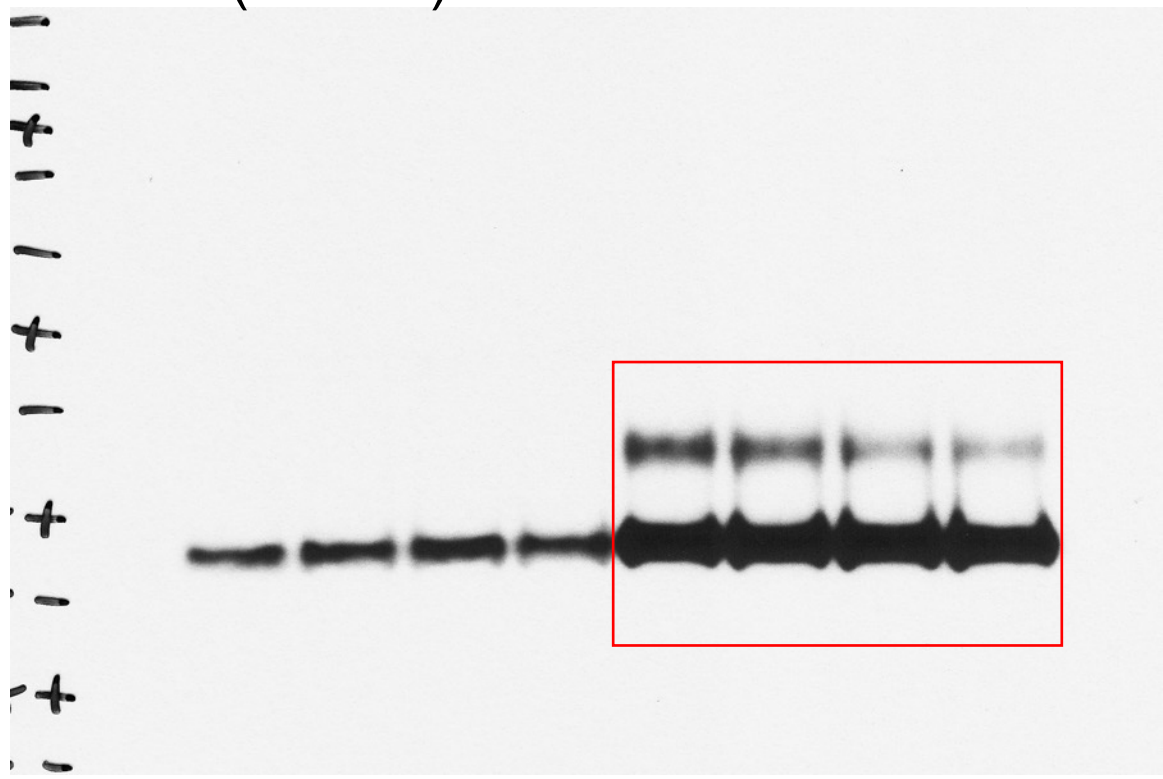

IB: P4HB

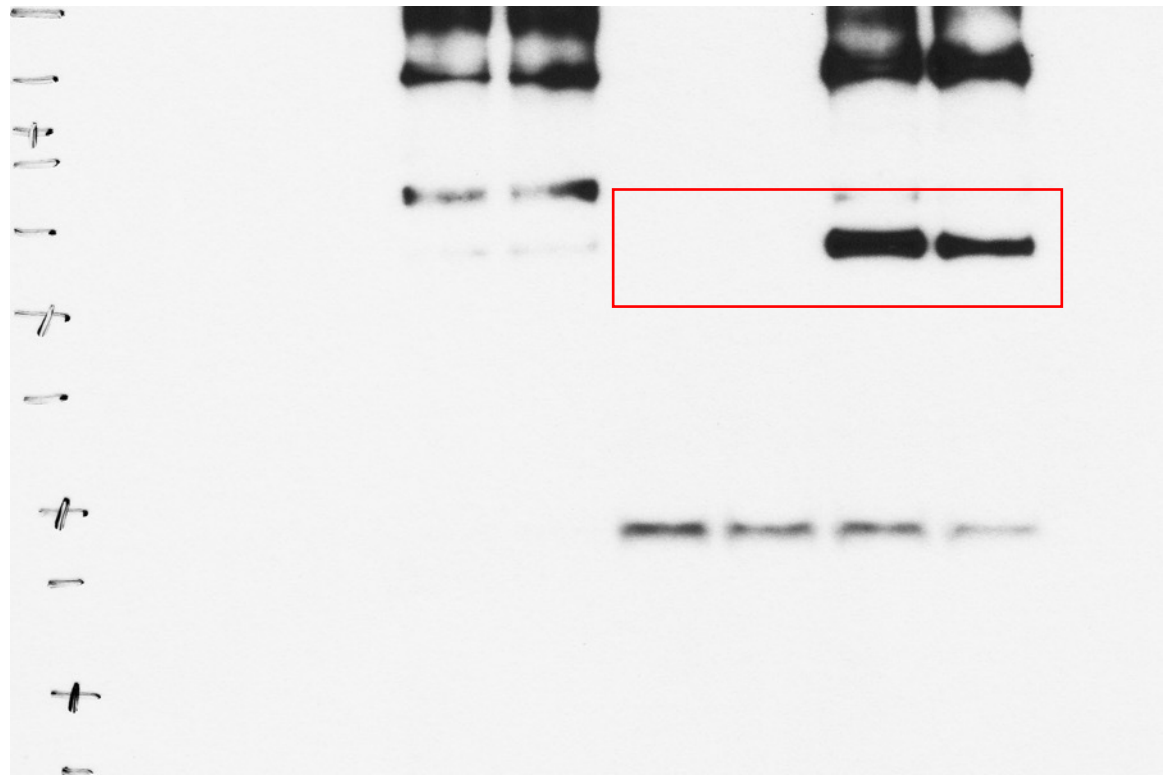

Fig 3

A

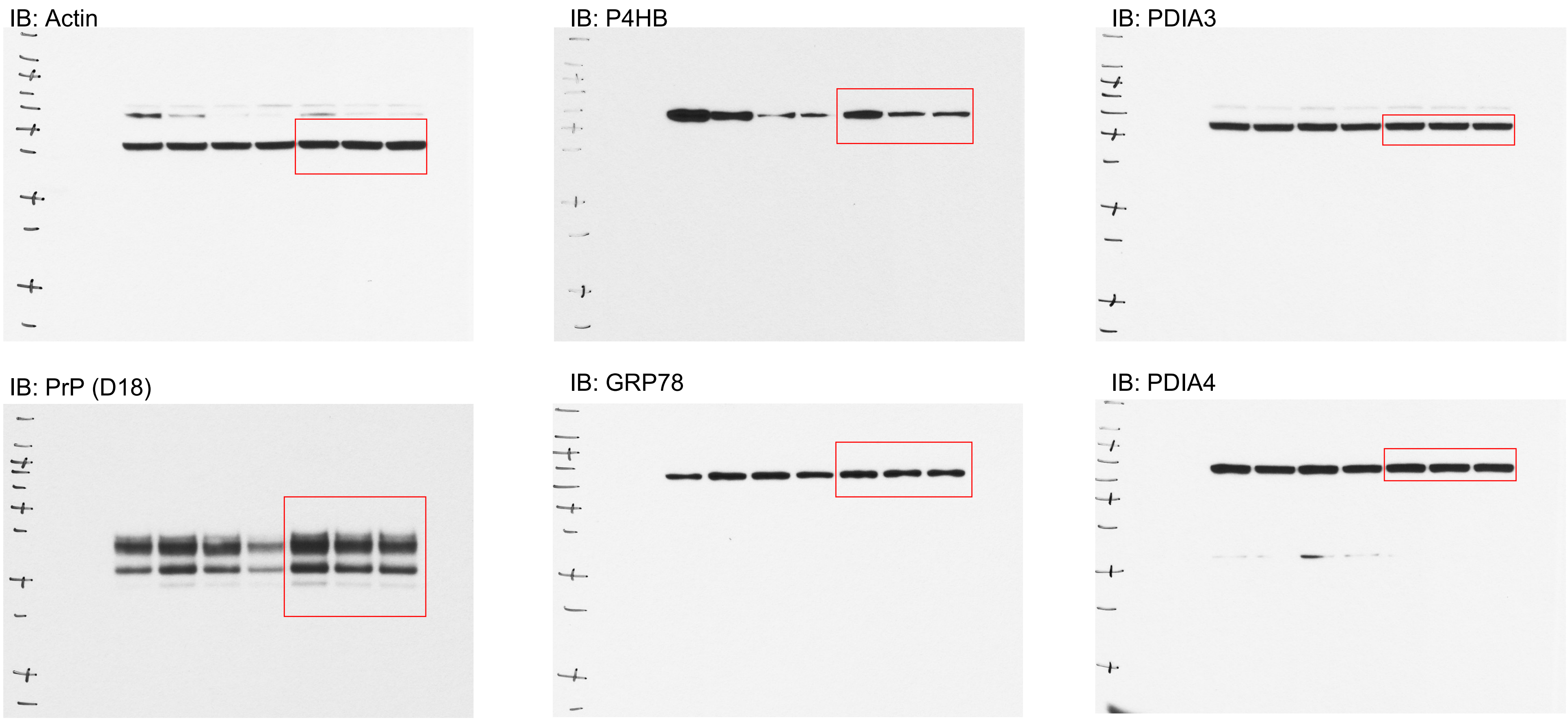

Fig 3

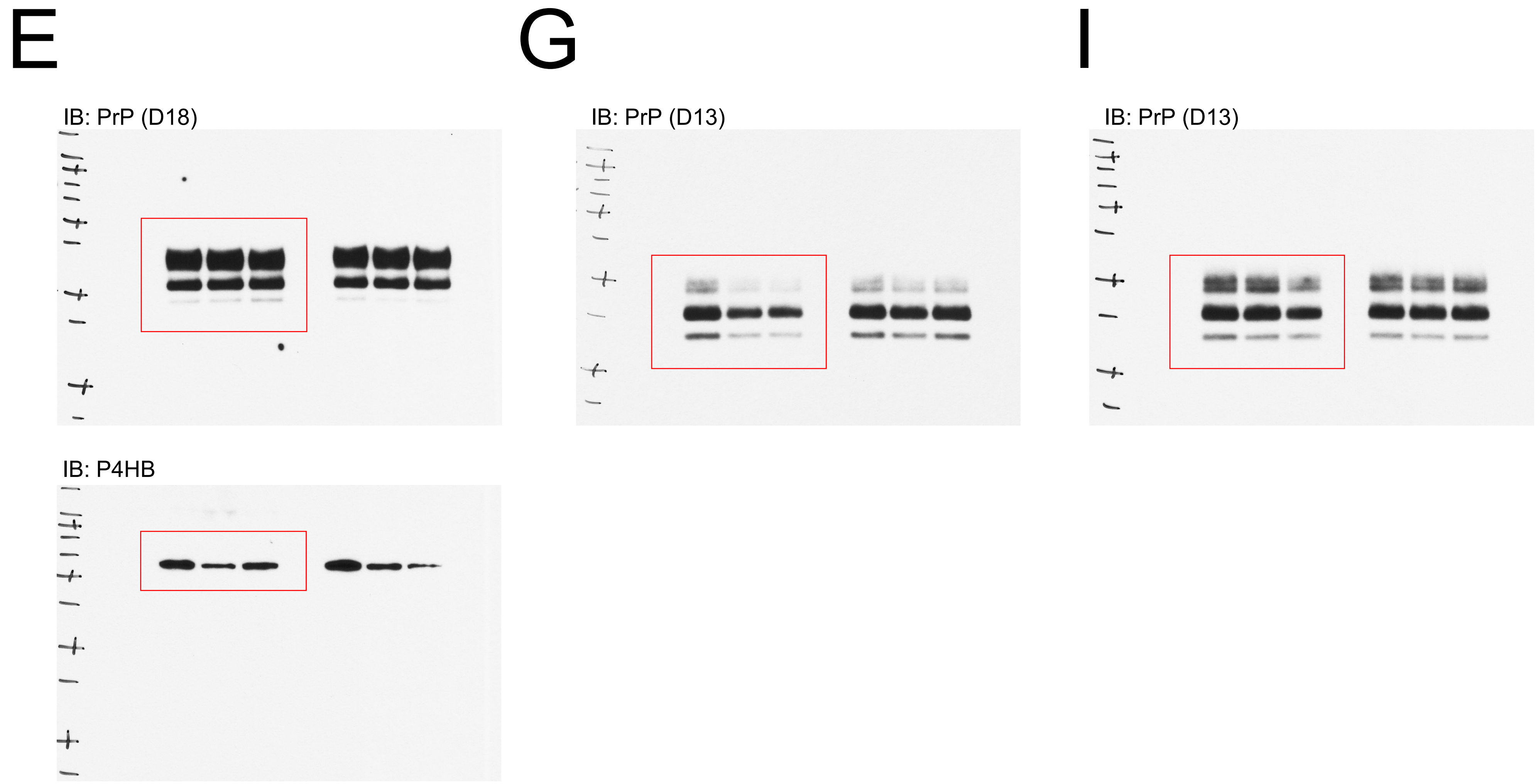

Fig 4

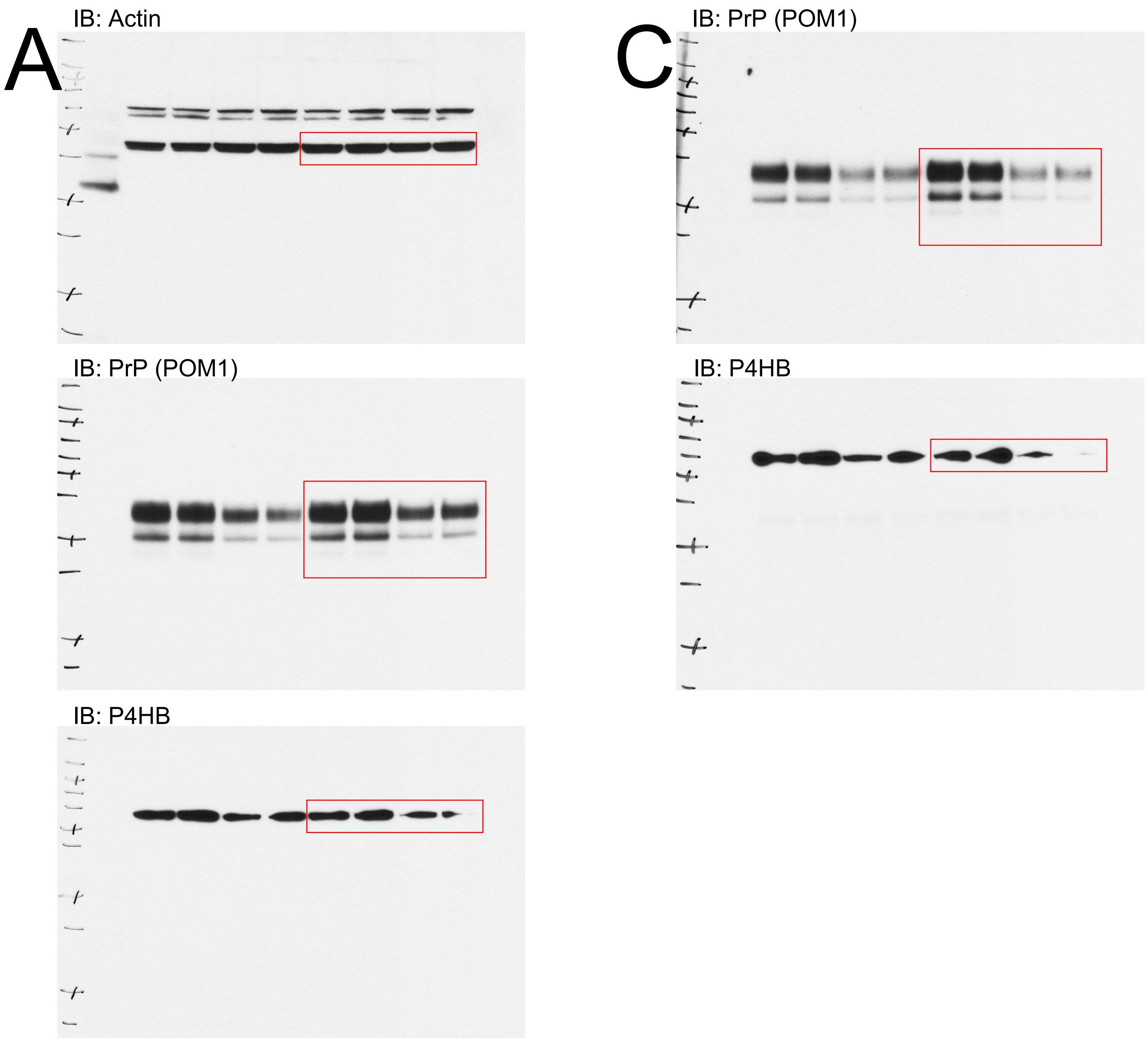

Fig 4

E (RML-infected)

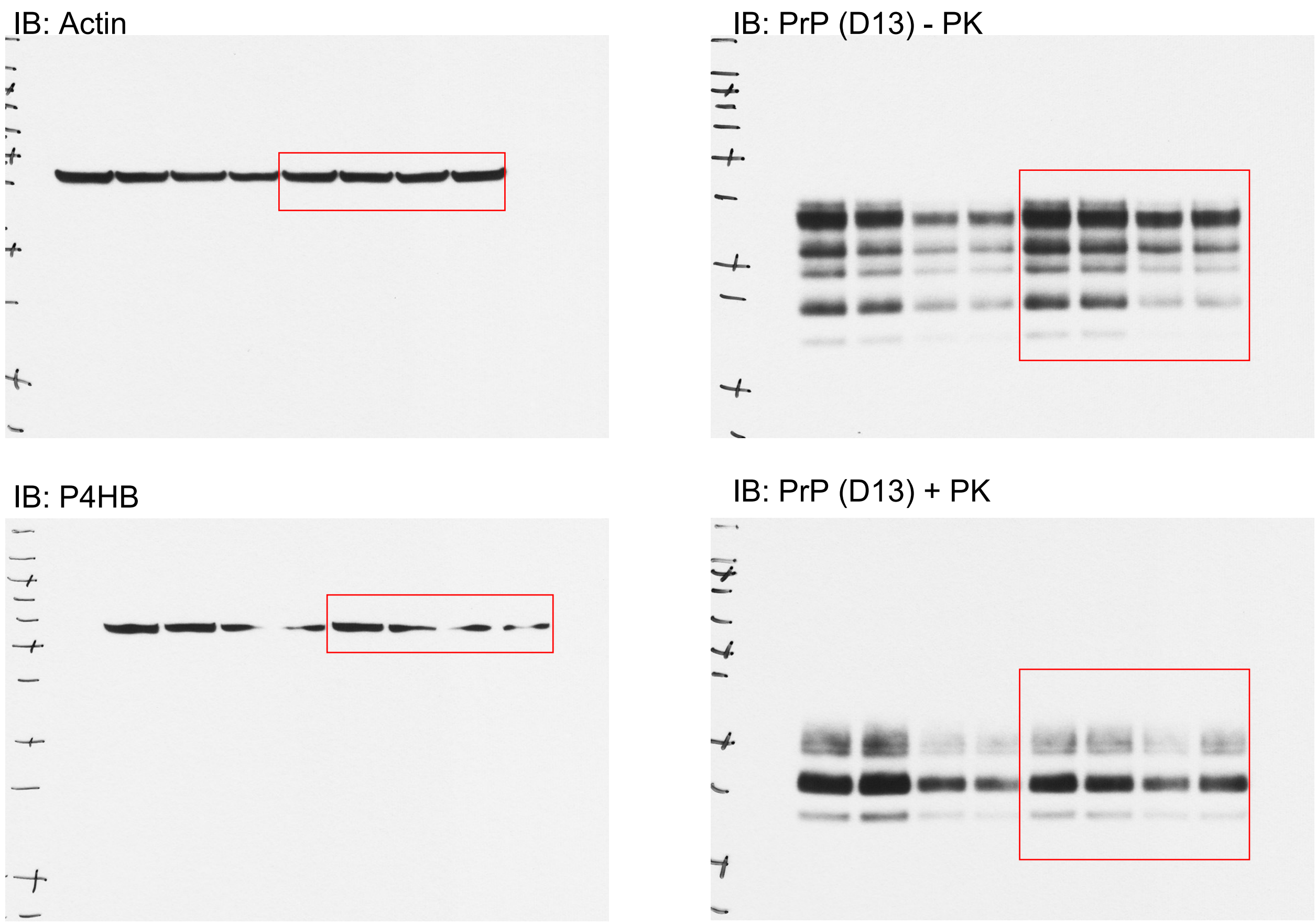

Fig 4

E (22L-infected)

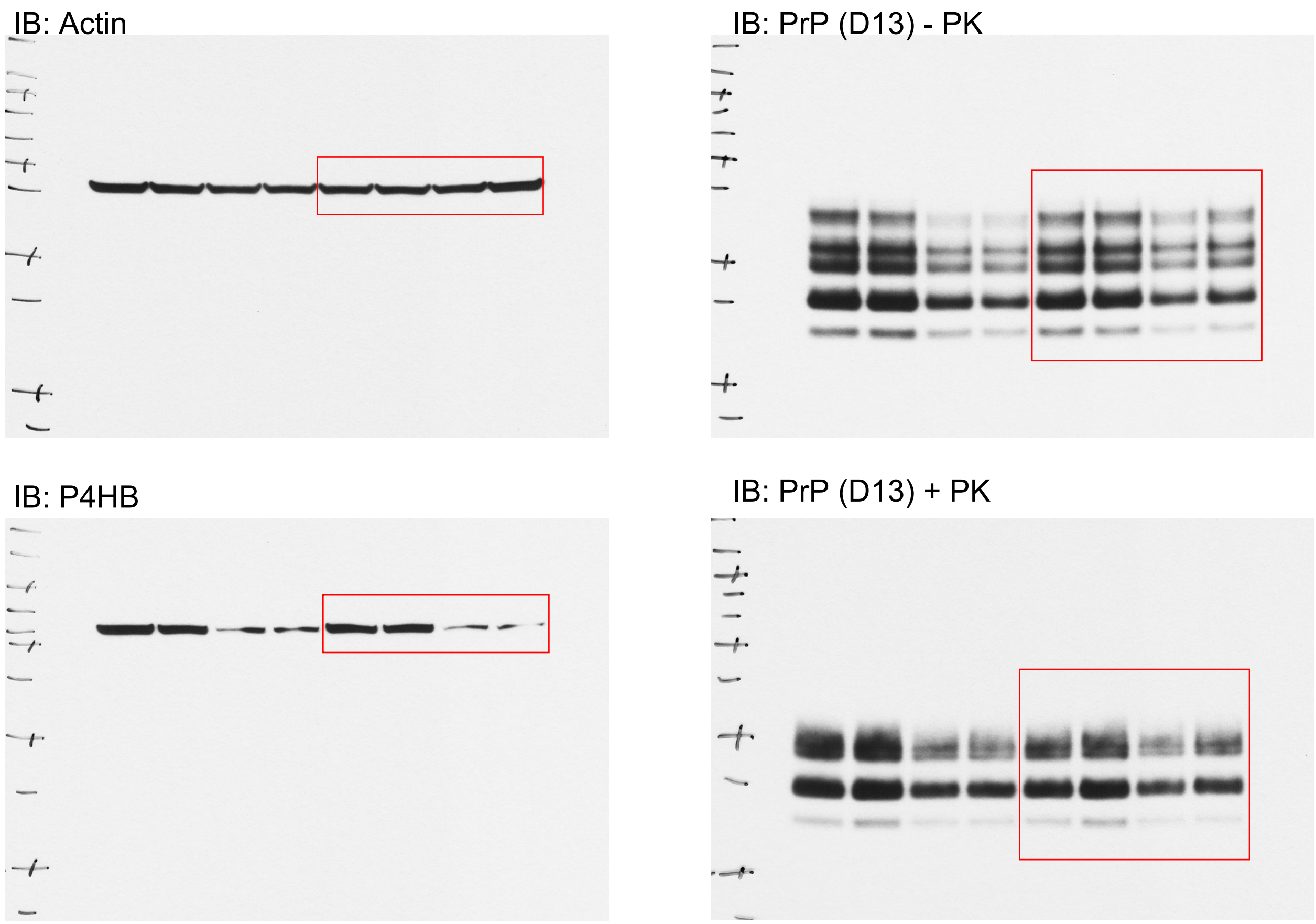

I

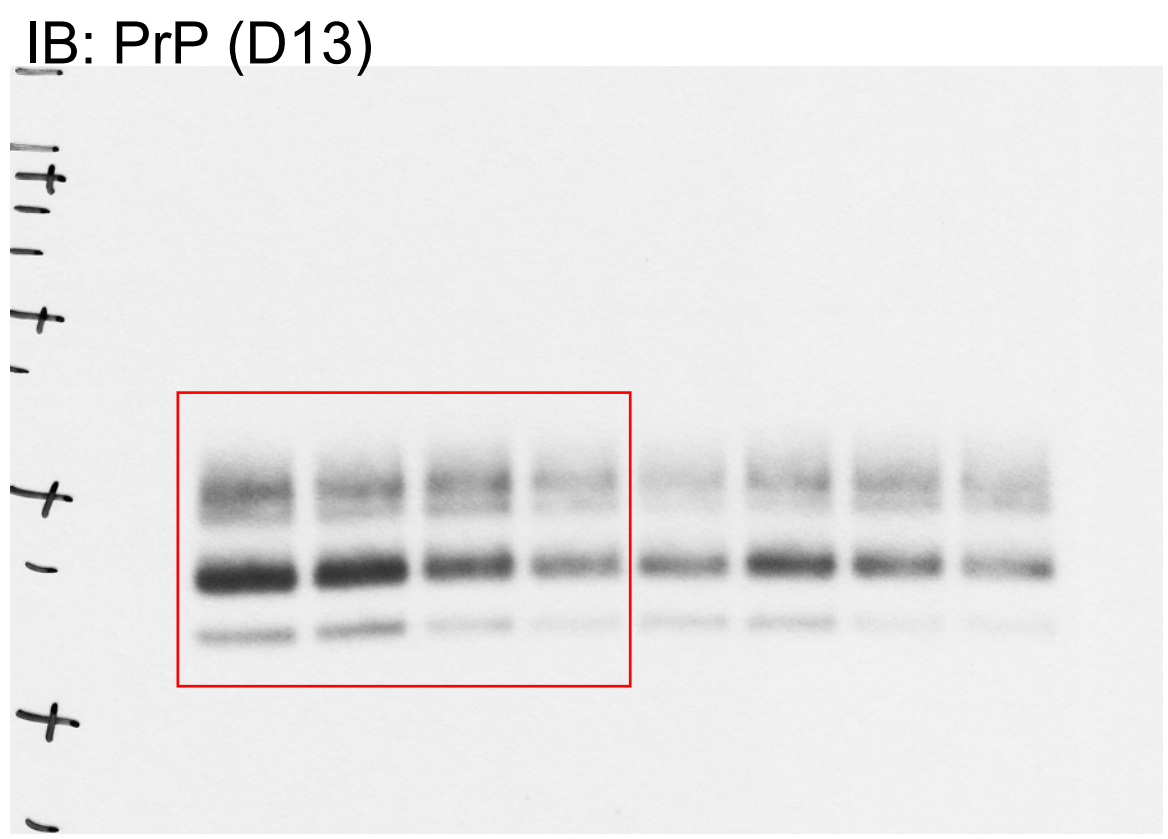

Fig 5

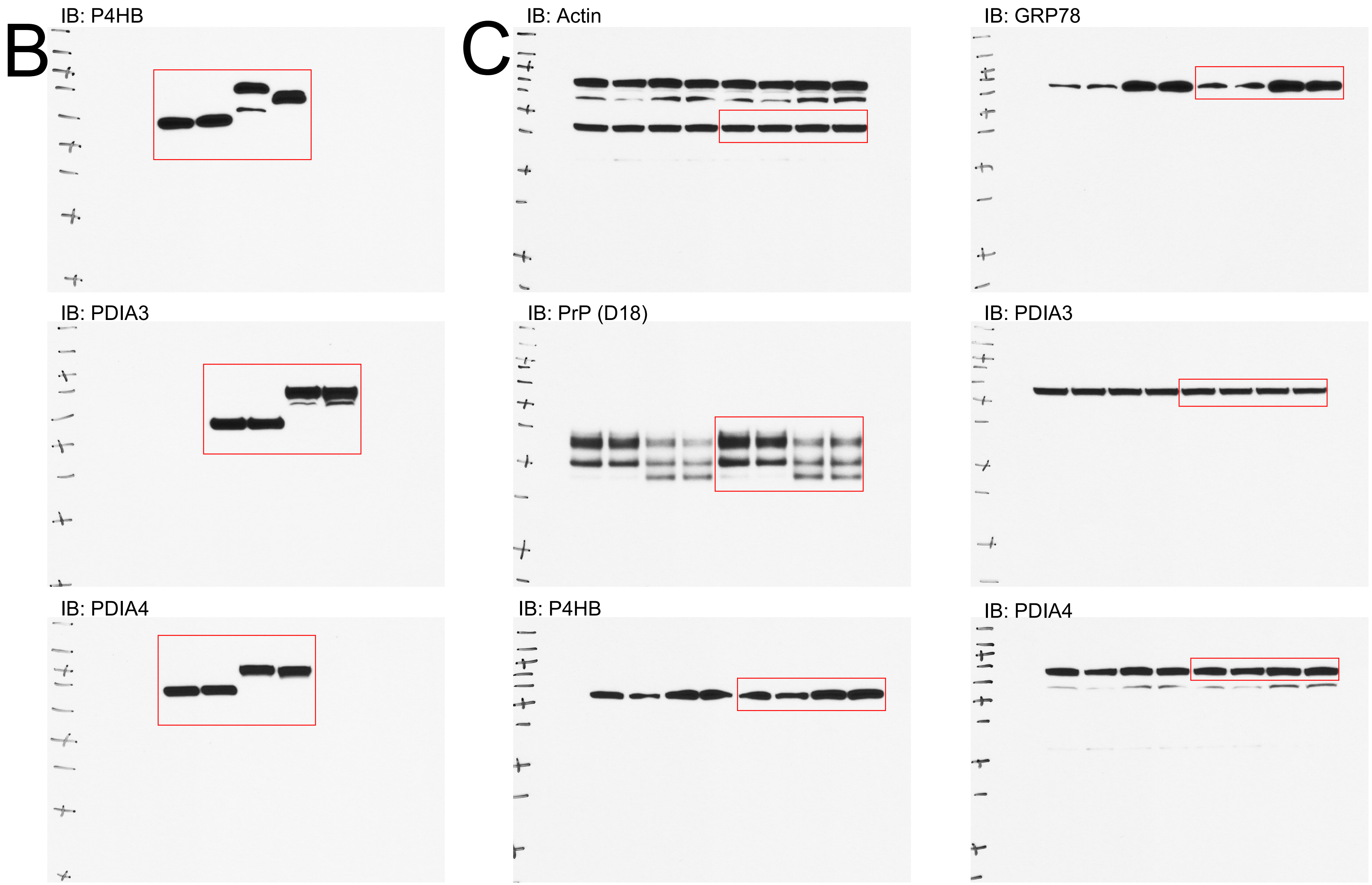
B
IB: P4HB
IB: PDIA3
IB: PDIA4
C
IB: Actin
IB: GRP78
IB: PrP (D18)
IB: PDIA3
IB: P4HB
IB: PDIA4

Fig 5

H

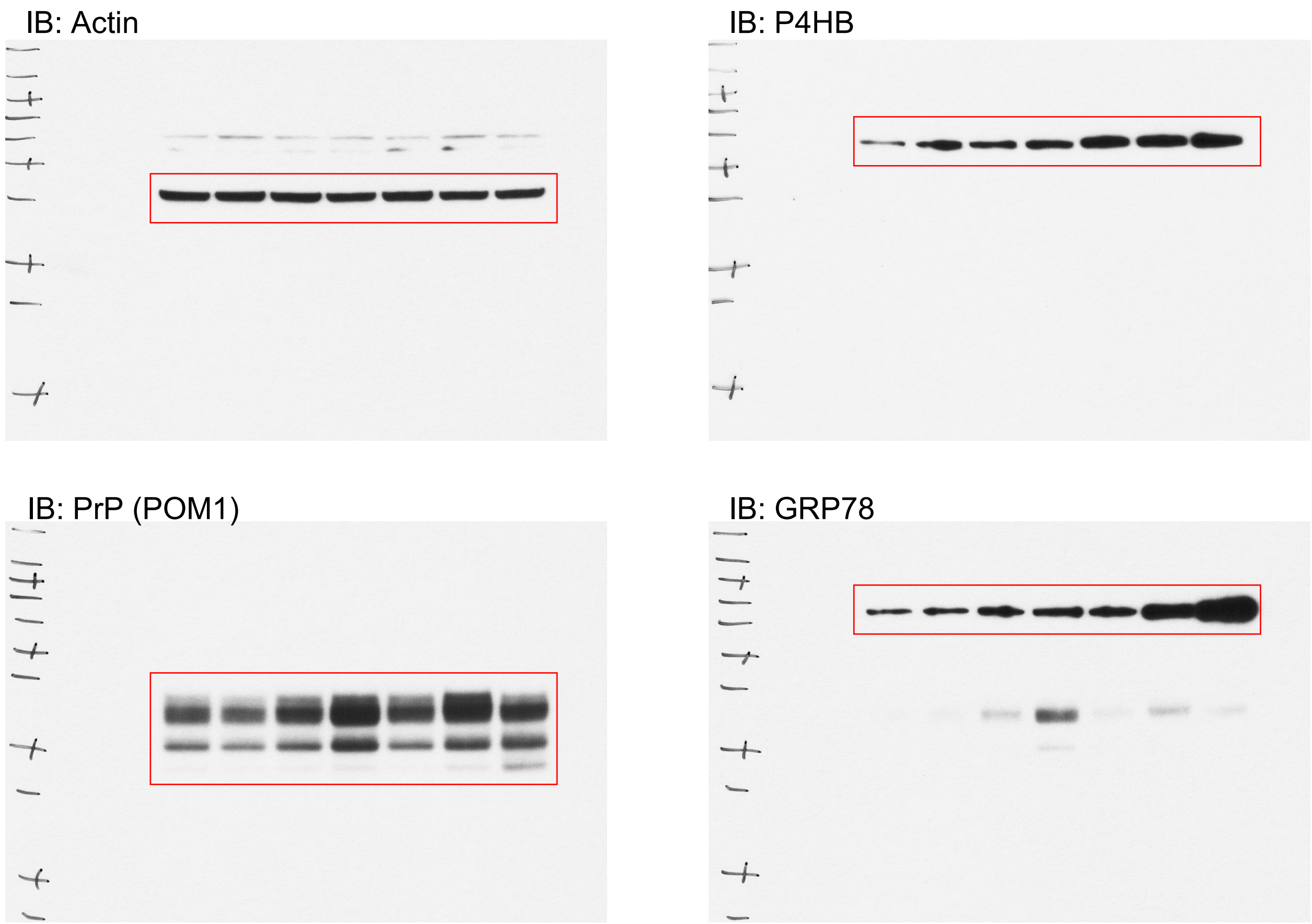

J

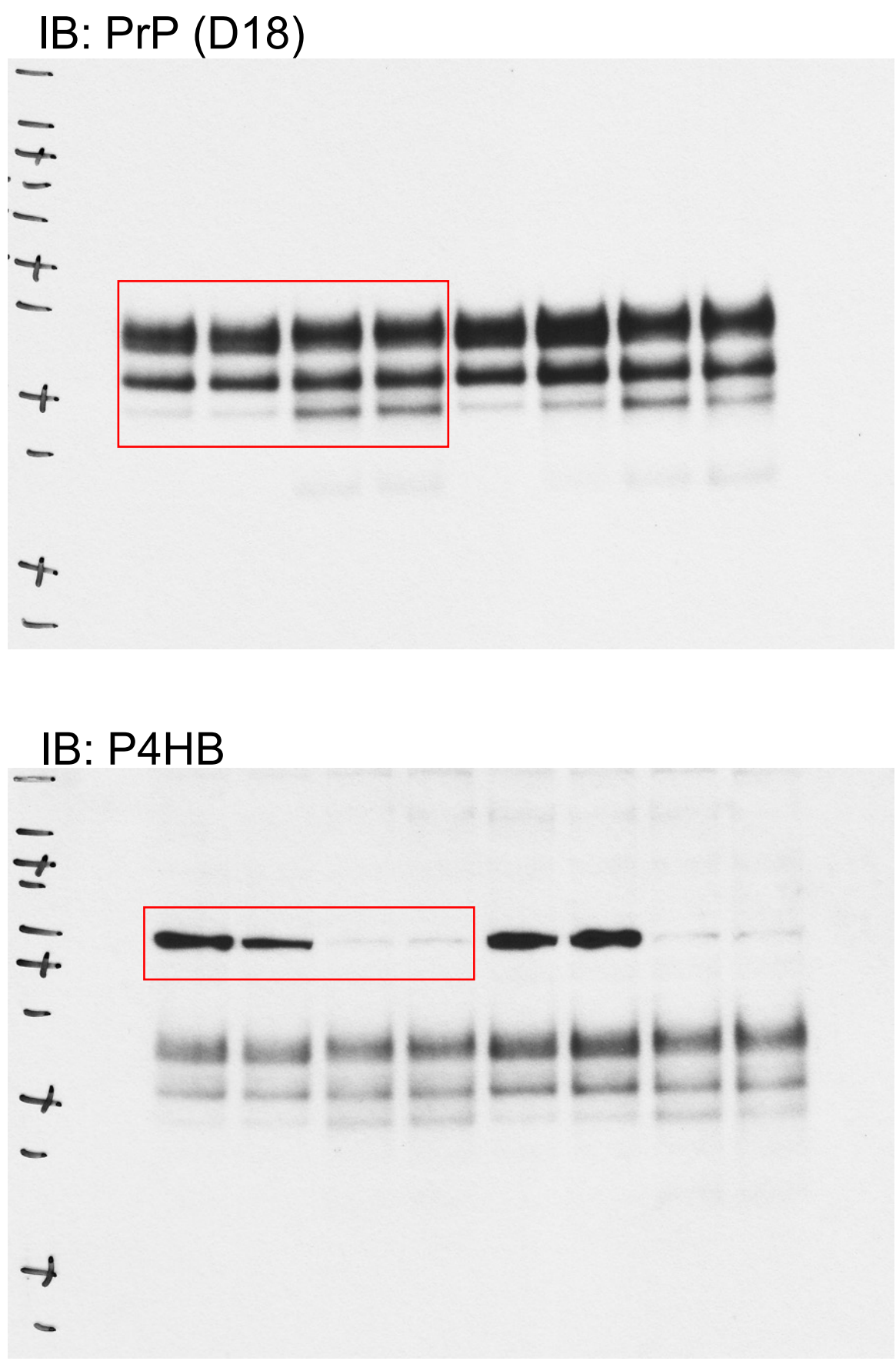

Fig 6

A (RML-infected CAD5)

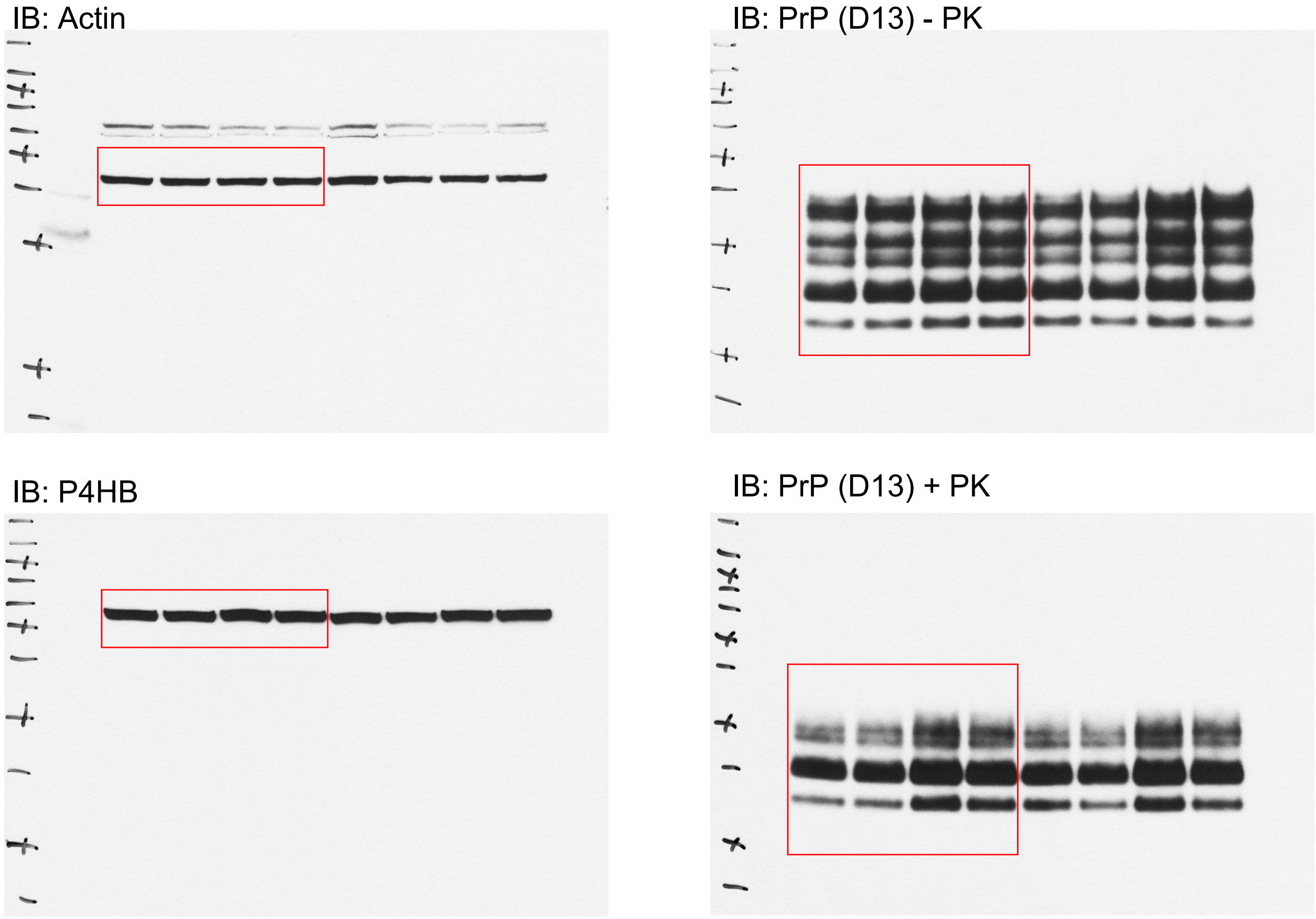

Fig 6

A (22L-infected CAD5)

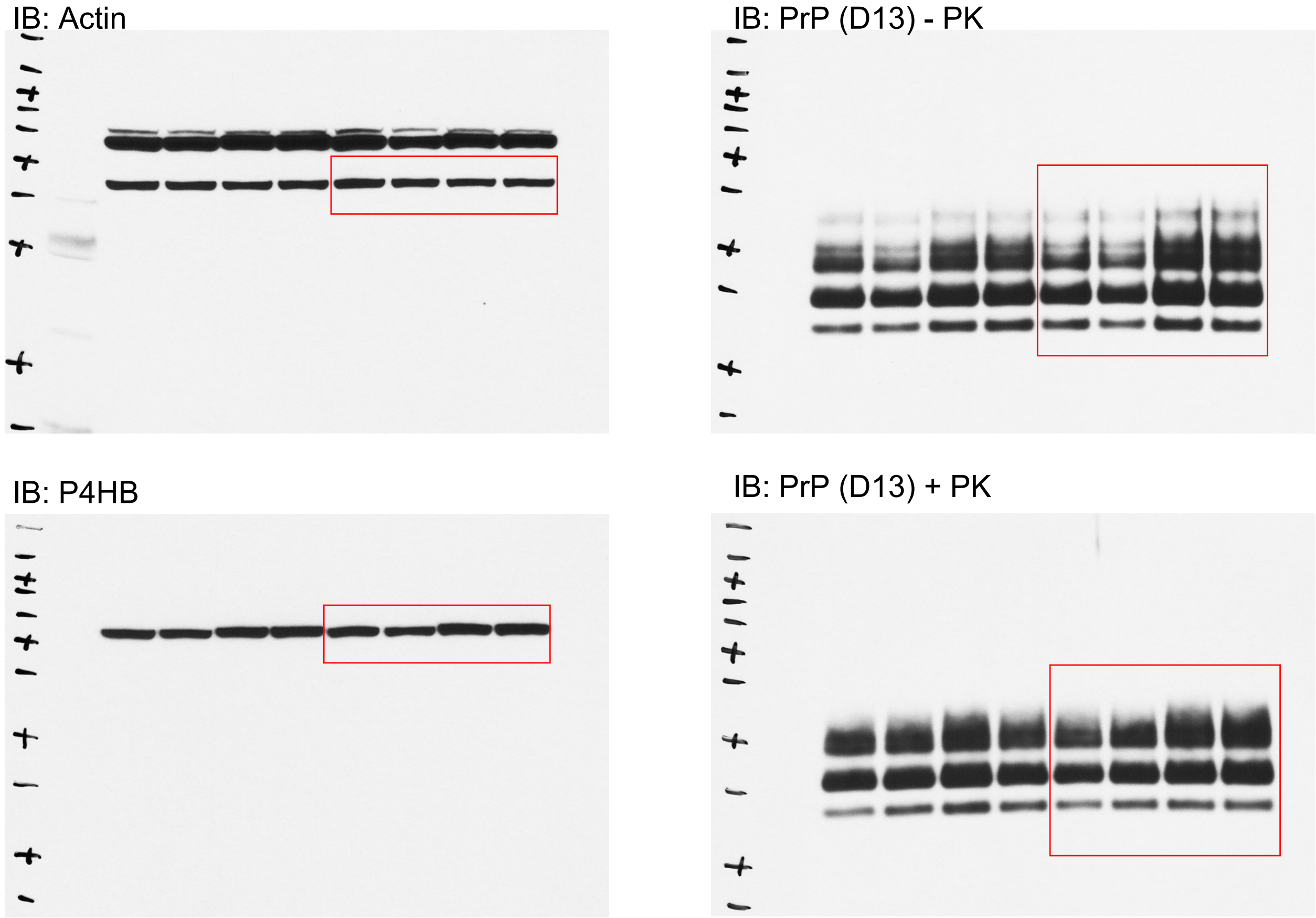

E

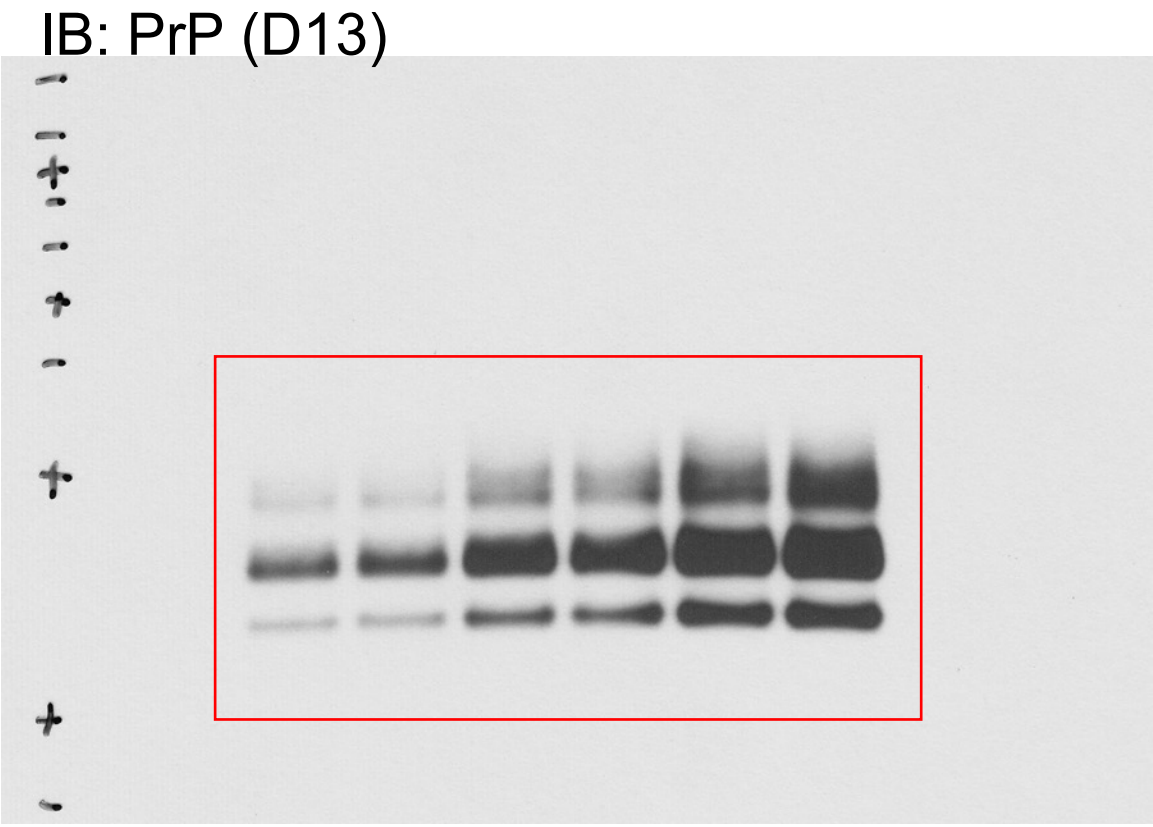

G

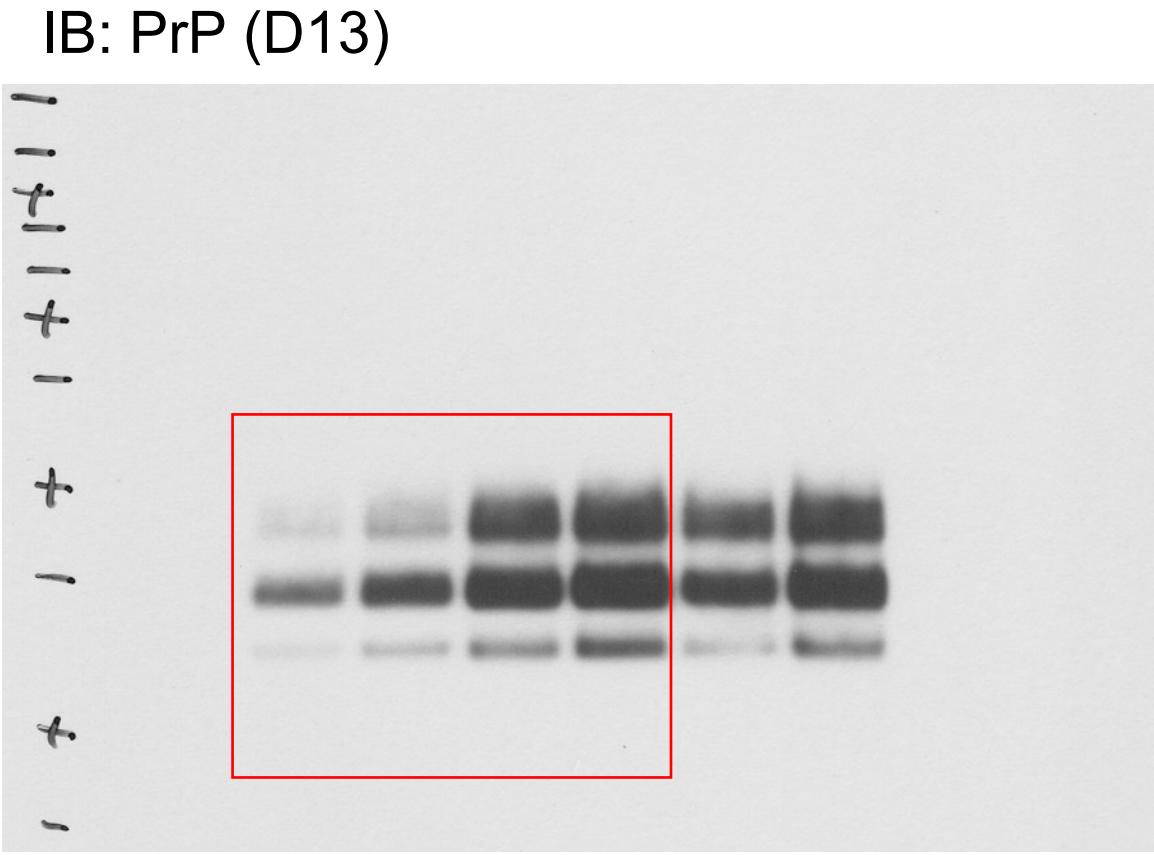

Fig 7

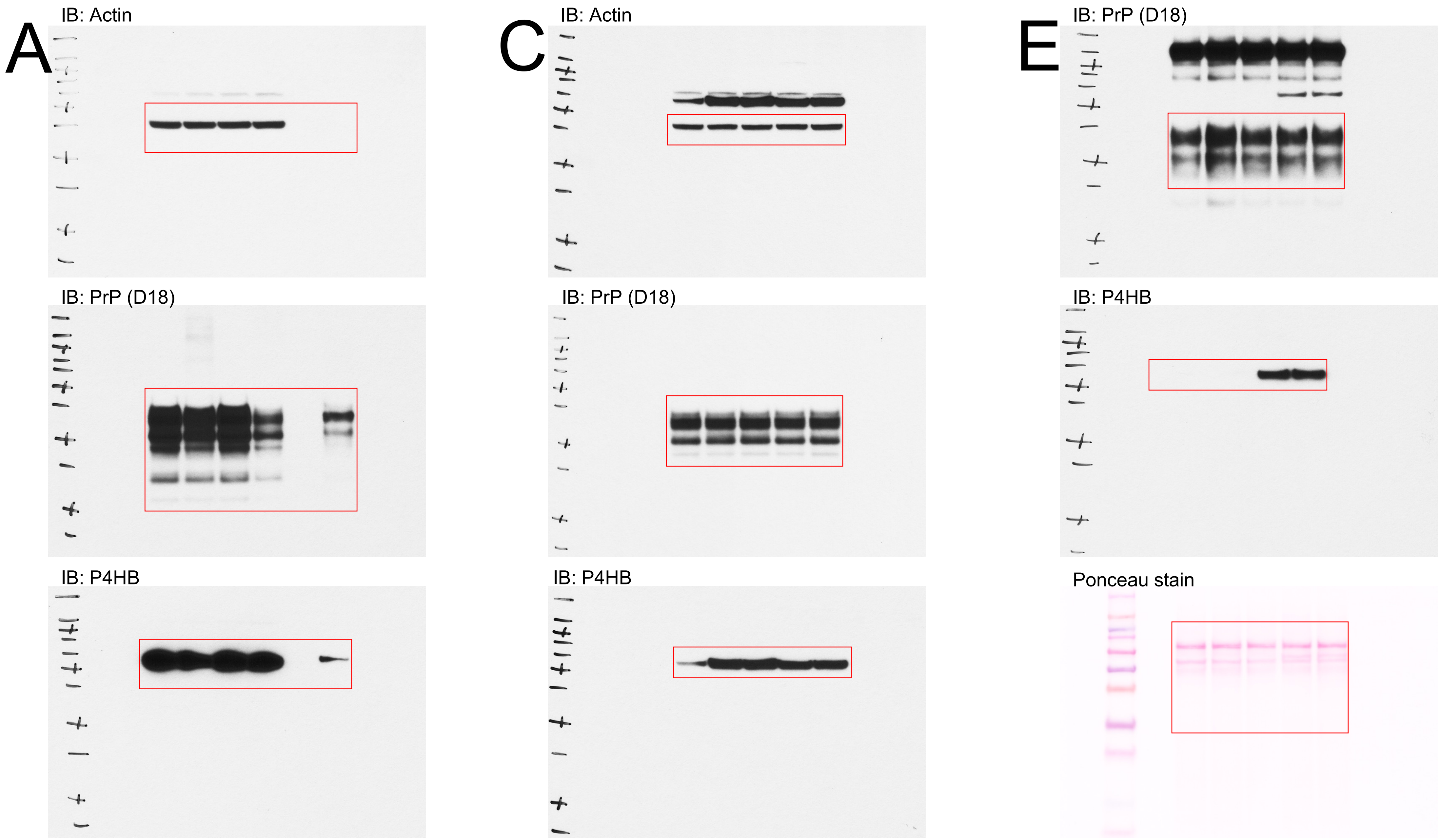
A
IB: Actin
IB: PrP (D18)
IB: P4HB
C
IB: Actin
IB: PrP (D18)
IB: P4HB
E
IB: PrP (D18)
IB: P4HB
Ponceau stain

Fig 7

F (RML-infected CAD5)

IB: PrP (D13)

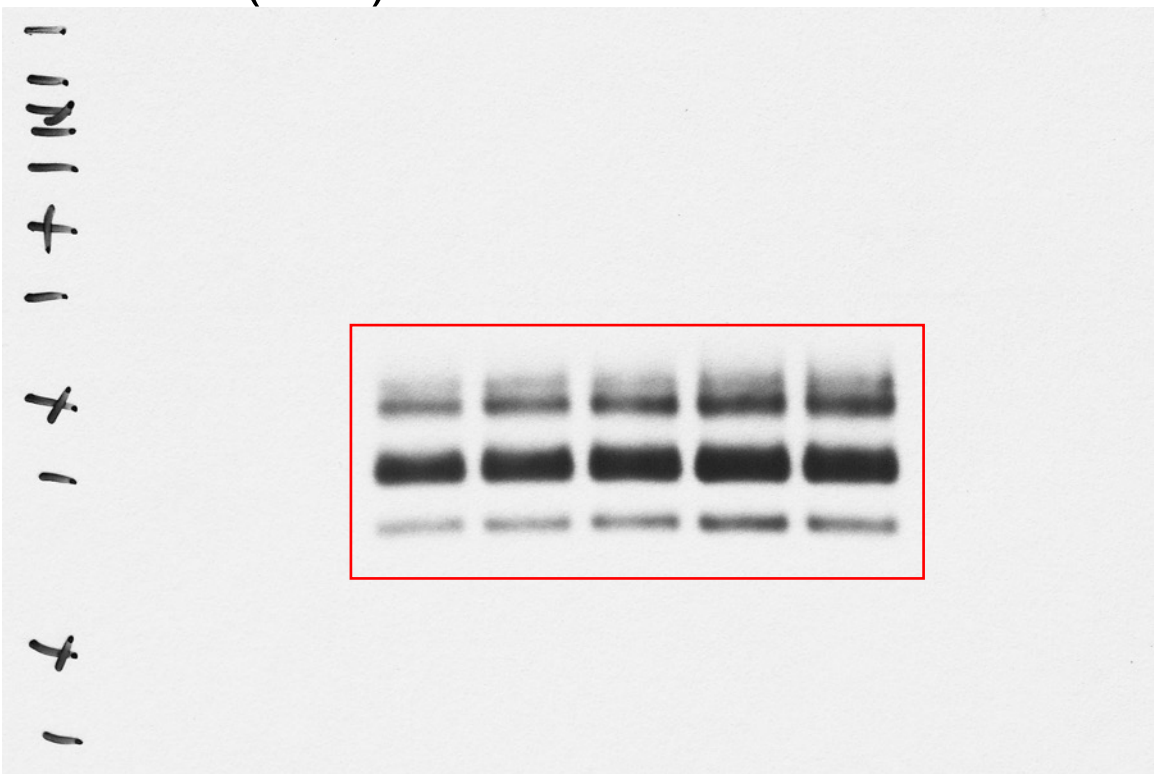

F (22L-infected CAD5)

IB: PrP (D13)

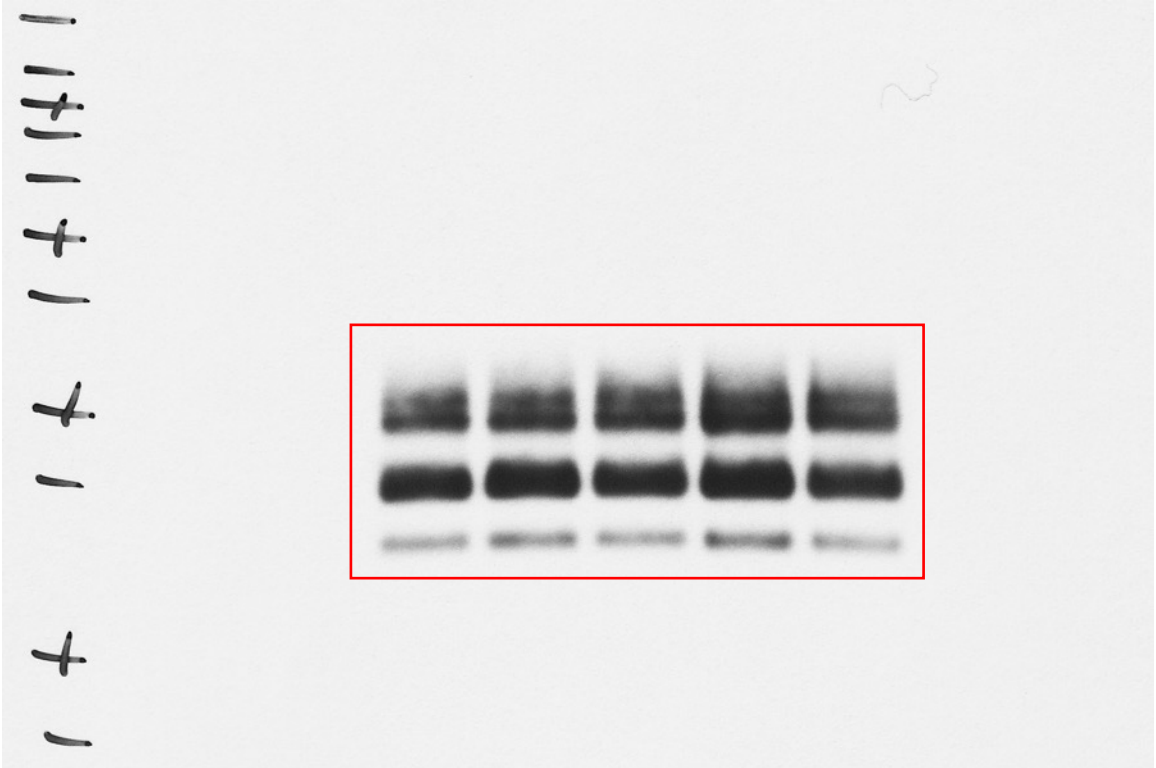

Fig 8

A

IB: P4HB

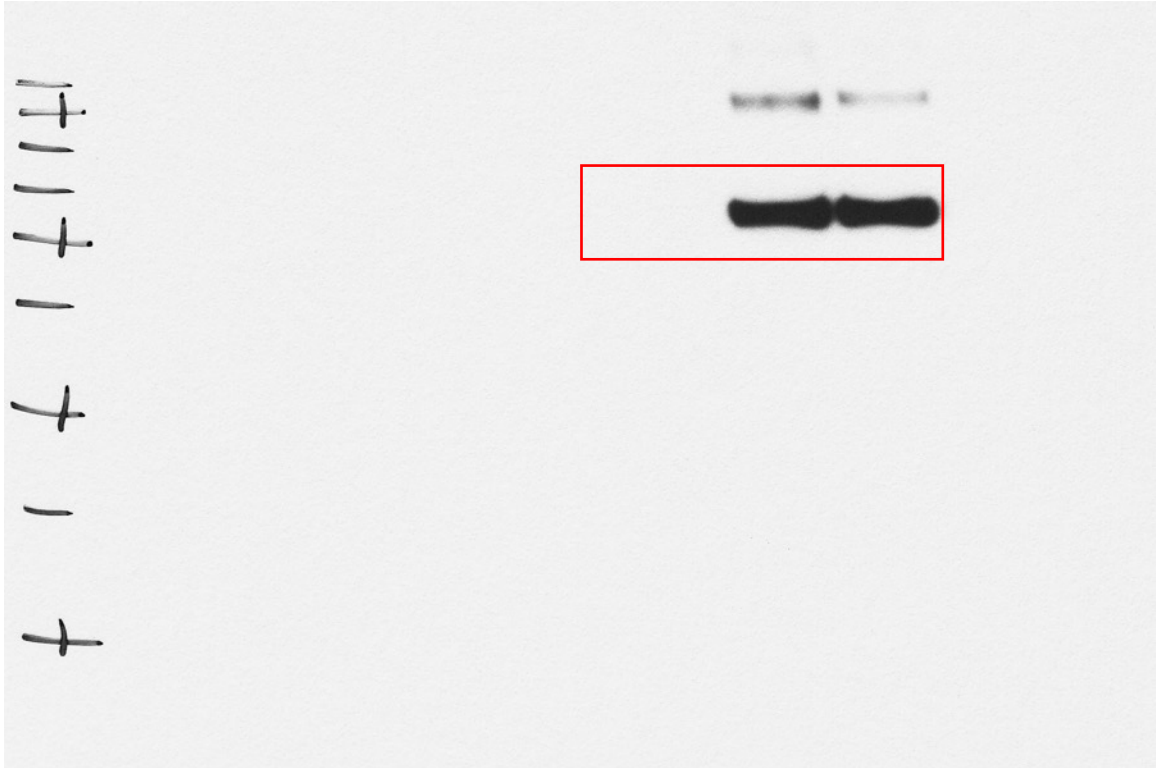

Ponceau stain

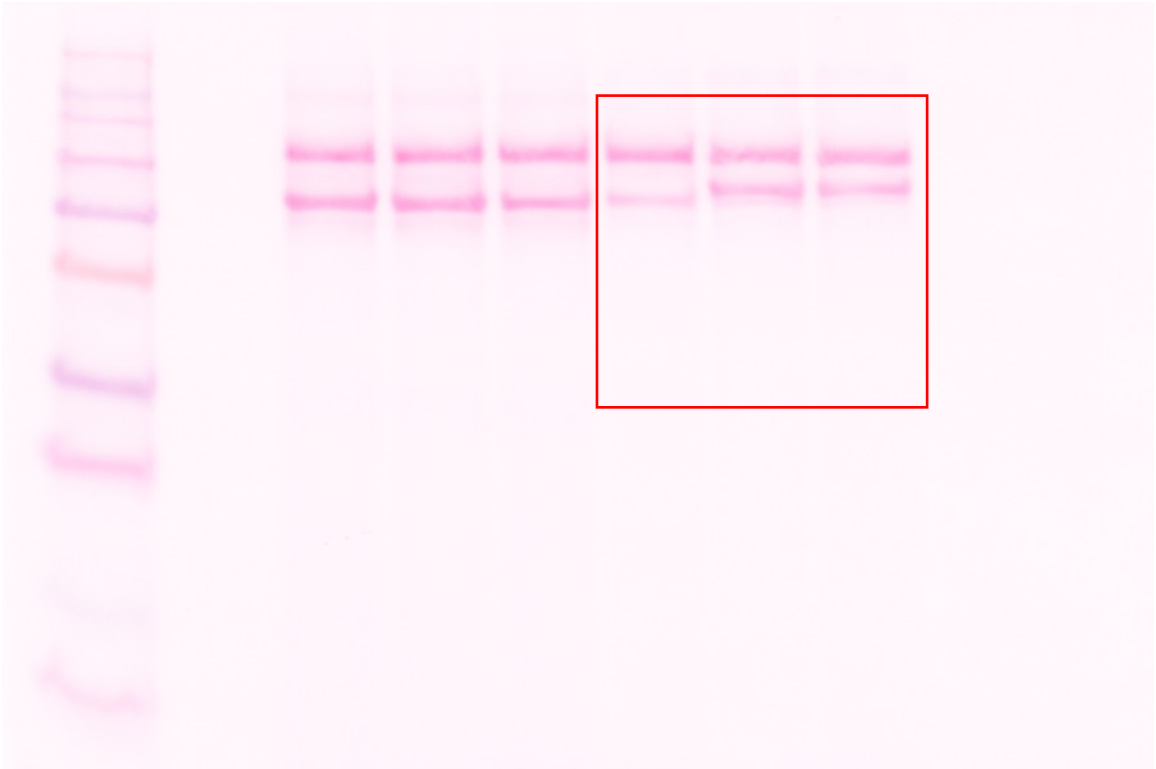

C

IB: PrP (D13)

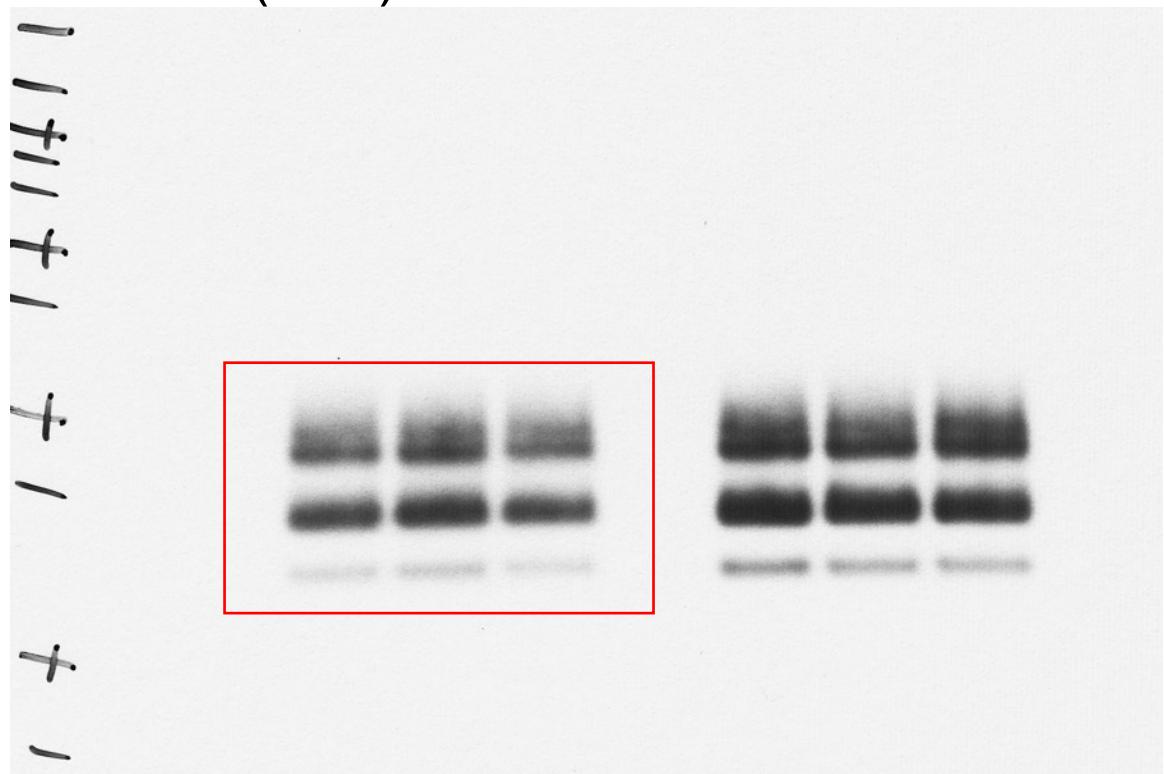

S1 Fig

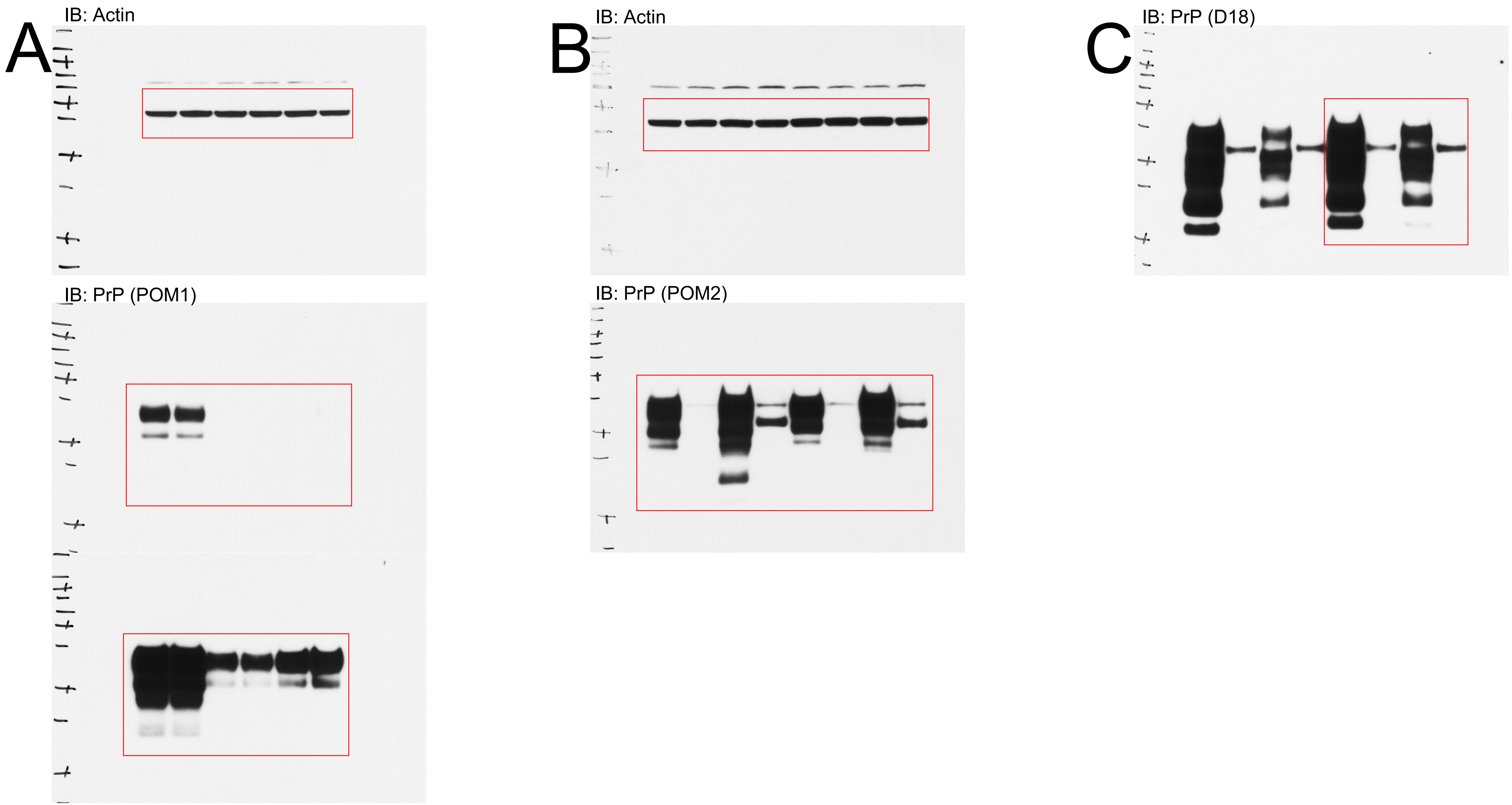

S2 Fig

A

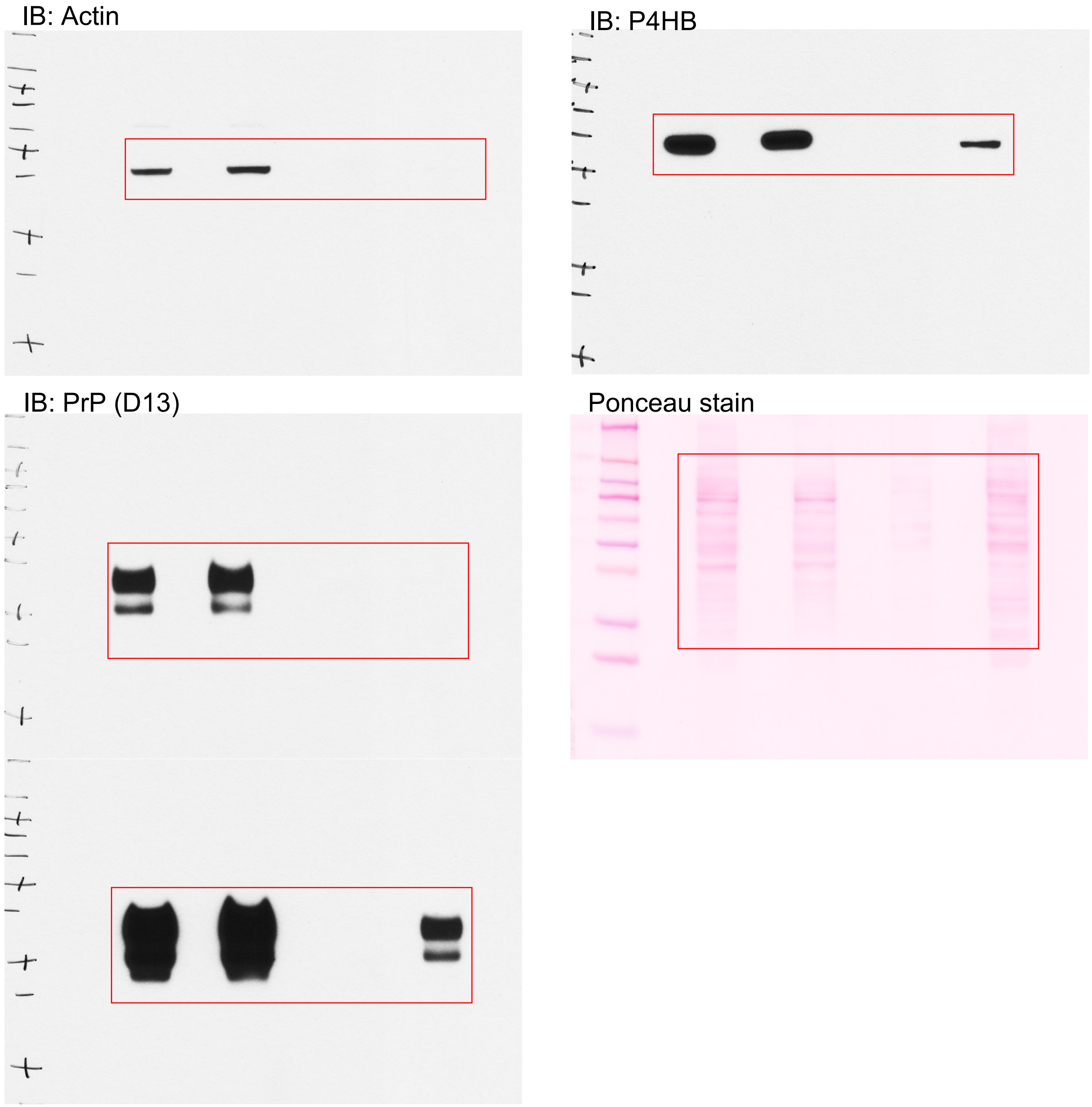

S3 Fig

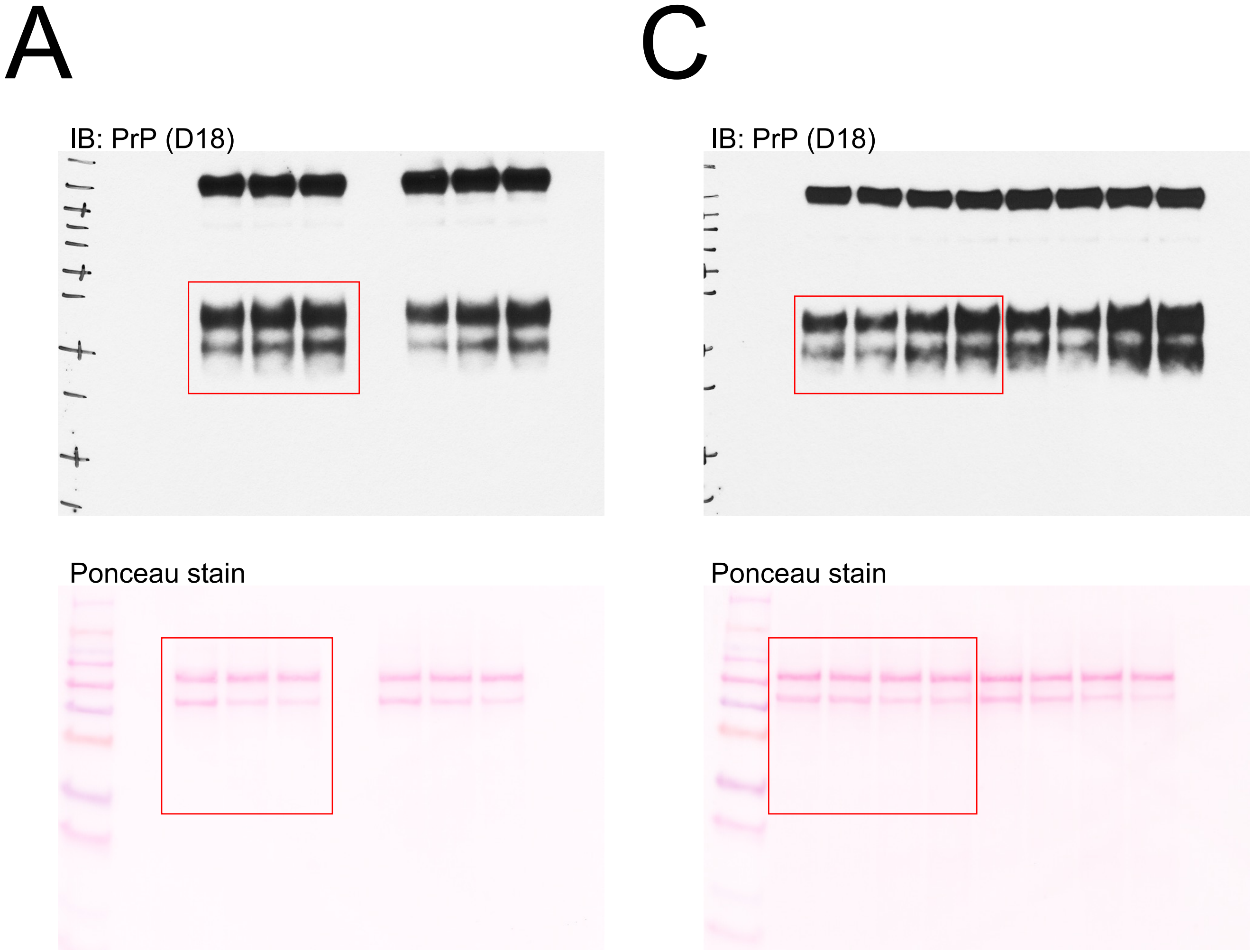

S4 Fig

A

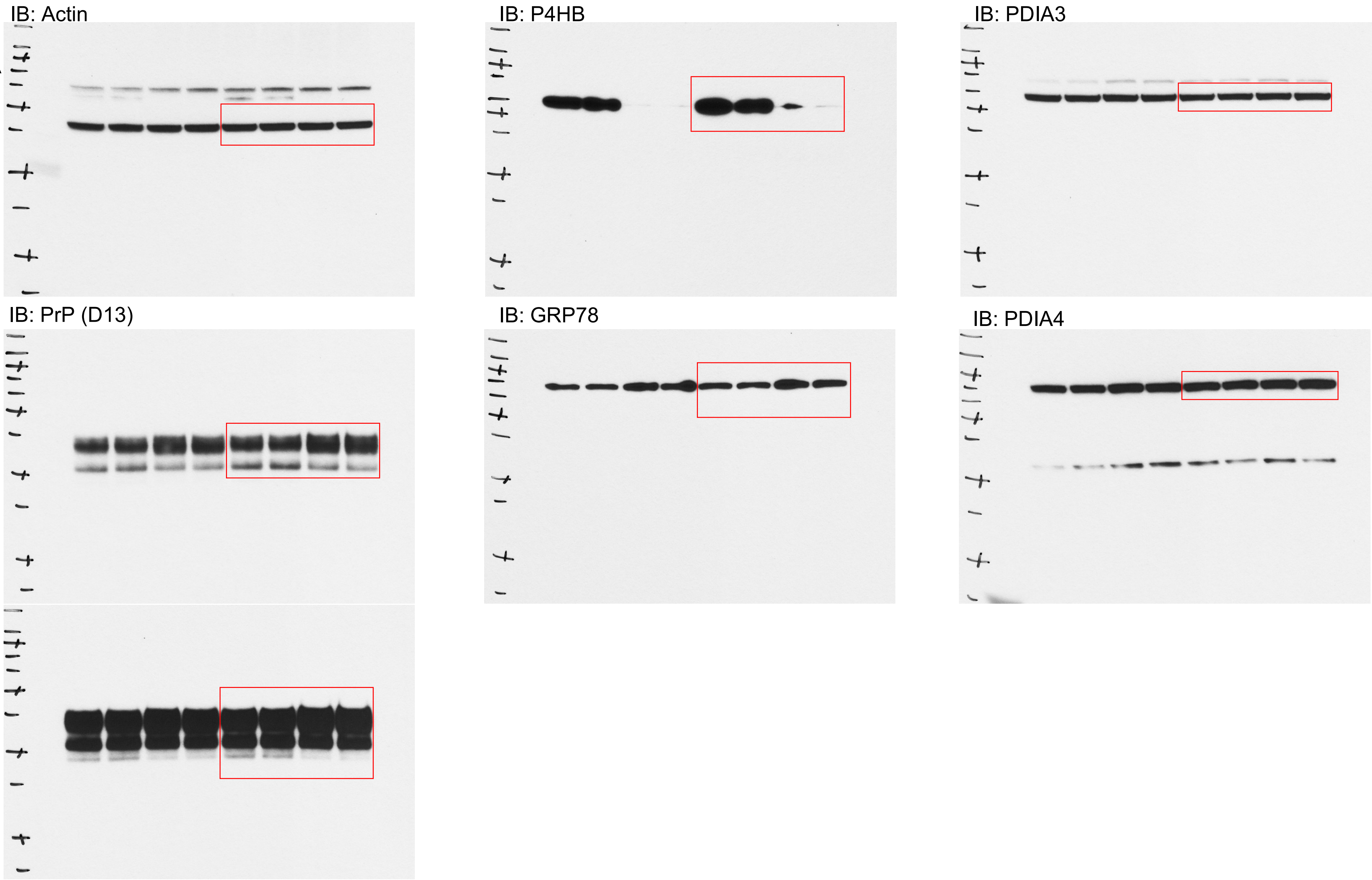

S4 Fig

E

IB: PrP (D18)

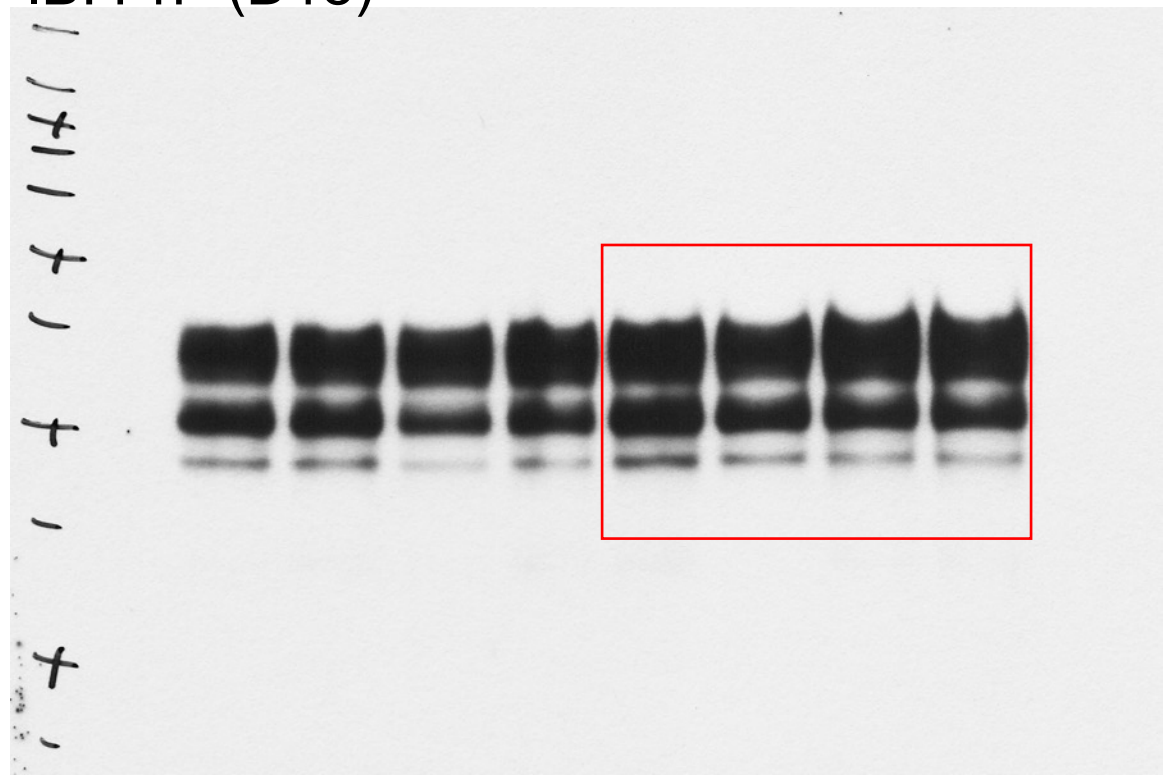

IB: P4HB

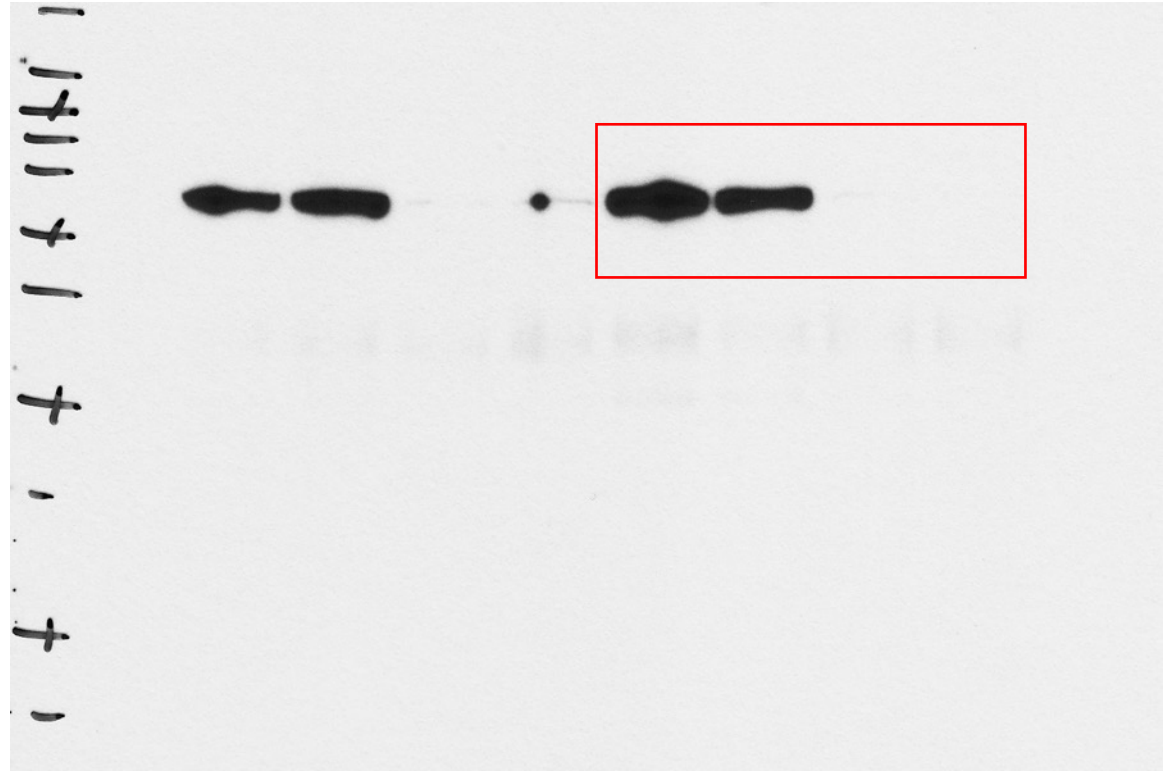

G

IB: PrP (D13)

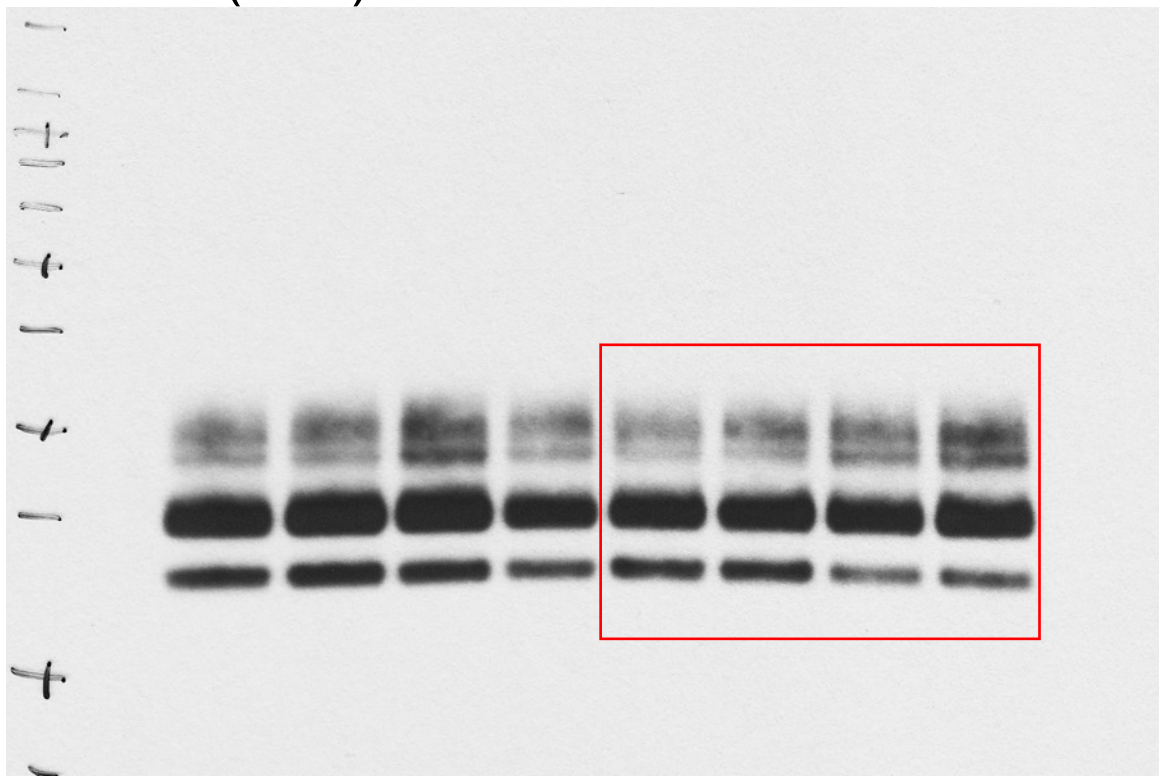

S5 Fig

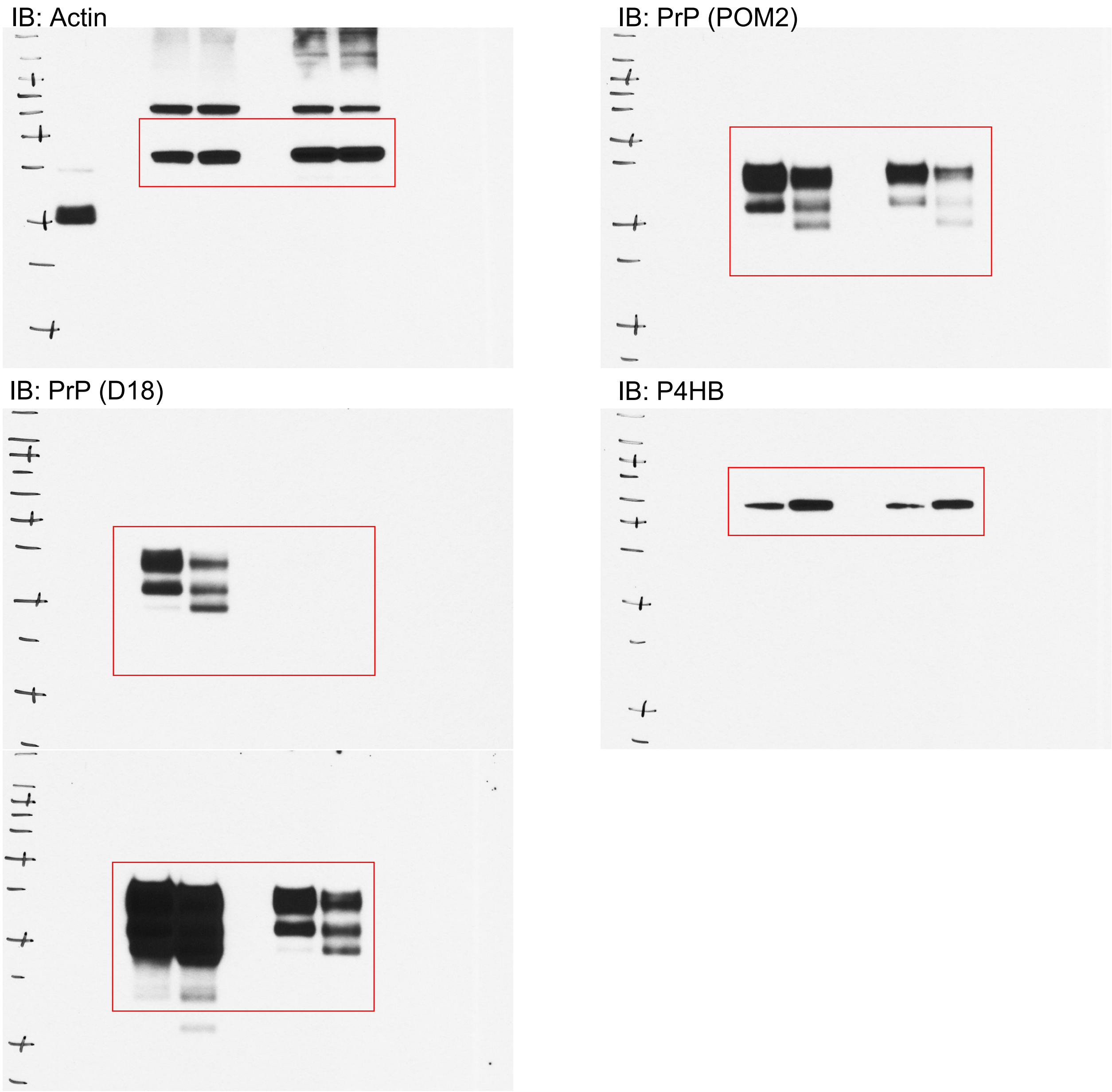

S6 Fig

A

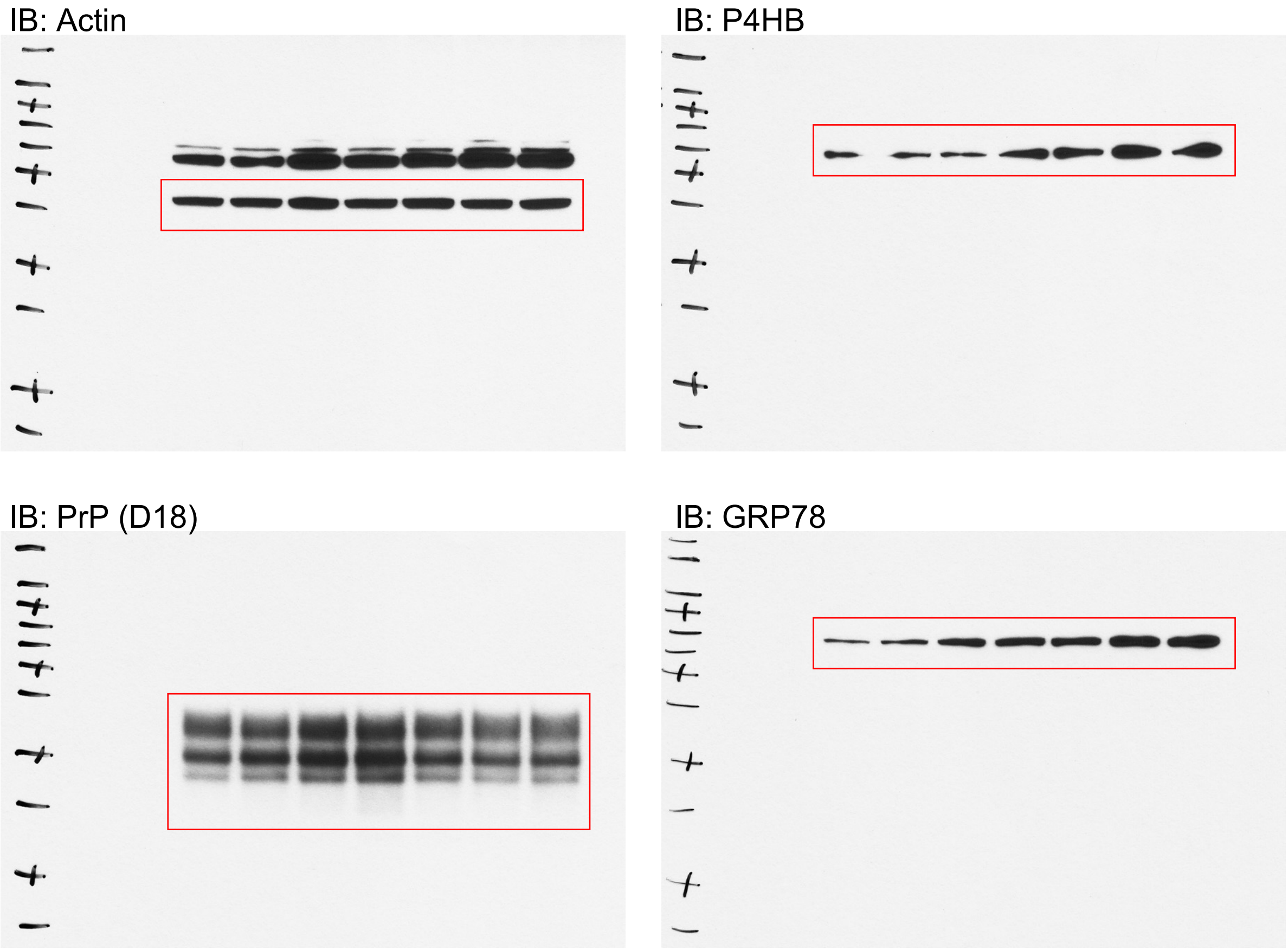

C

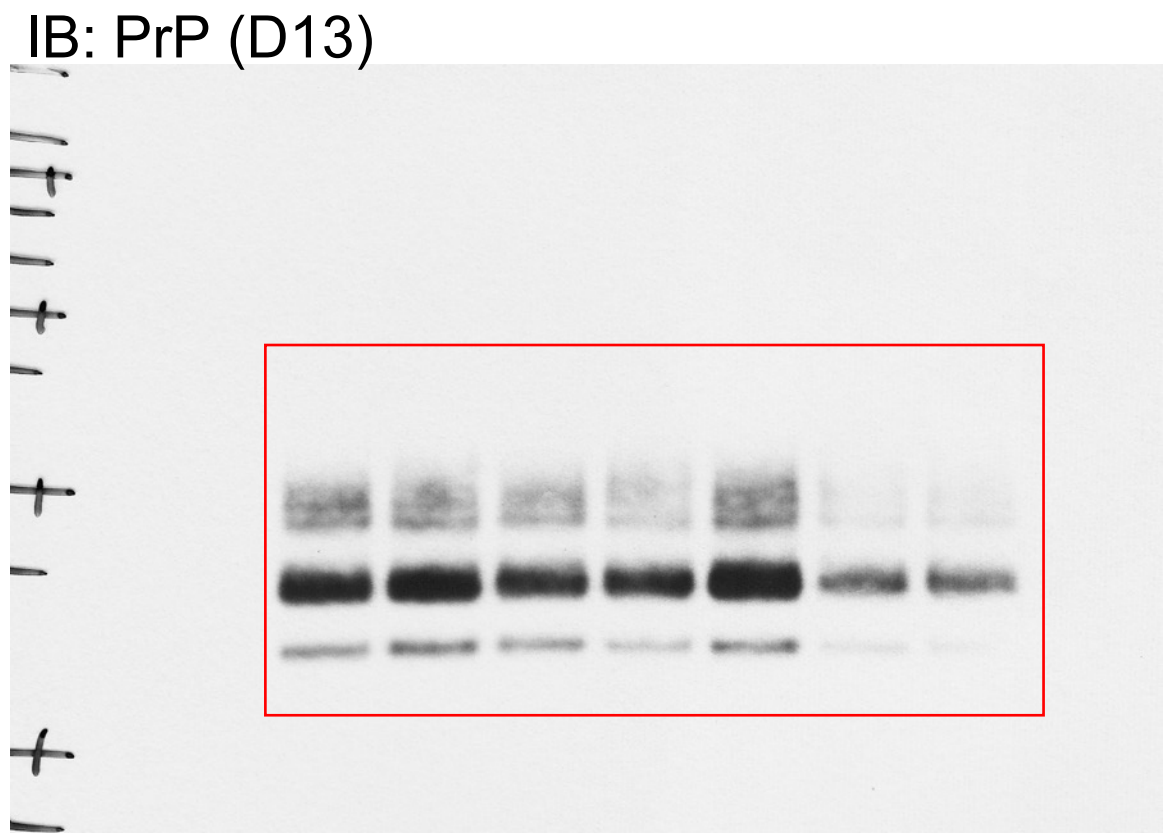

E

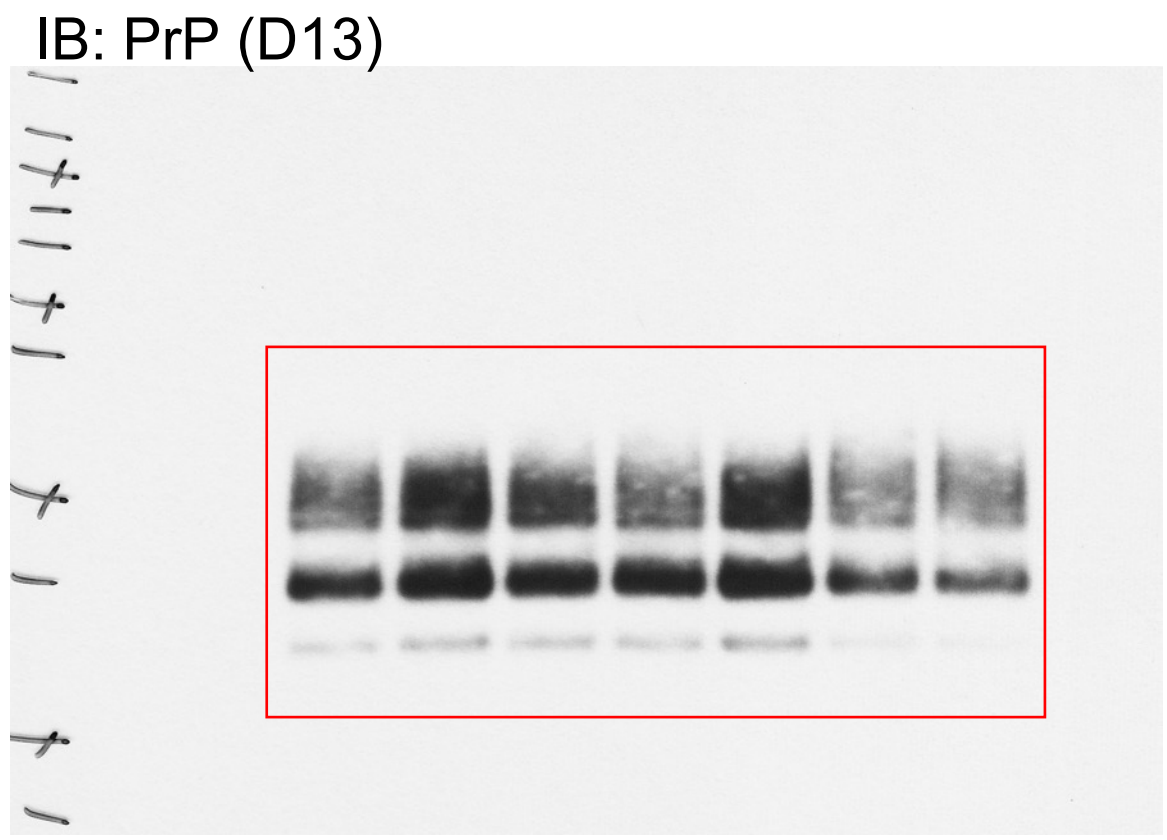

S7 Fig

A

IB: PrP (D18)

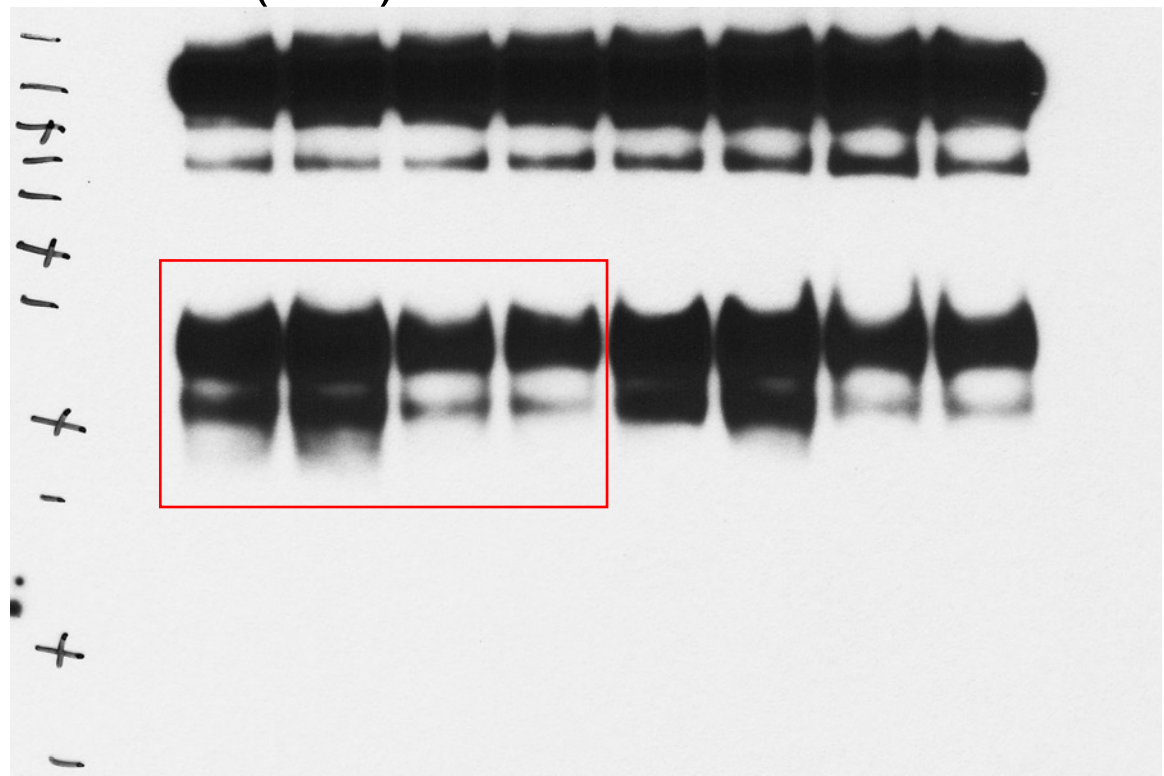

Ponceau stain

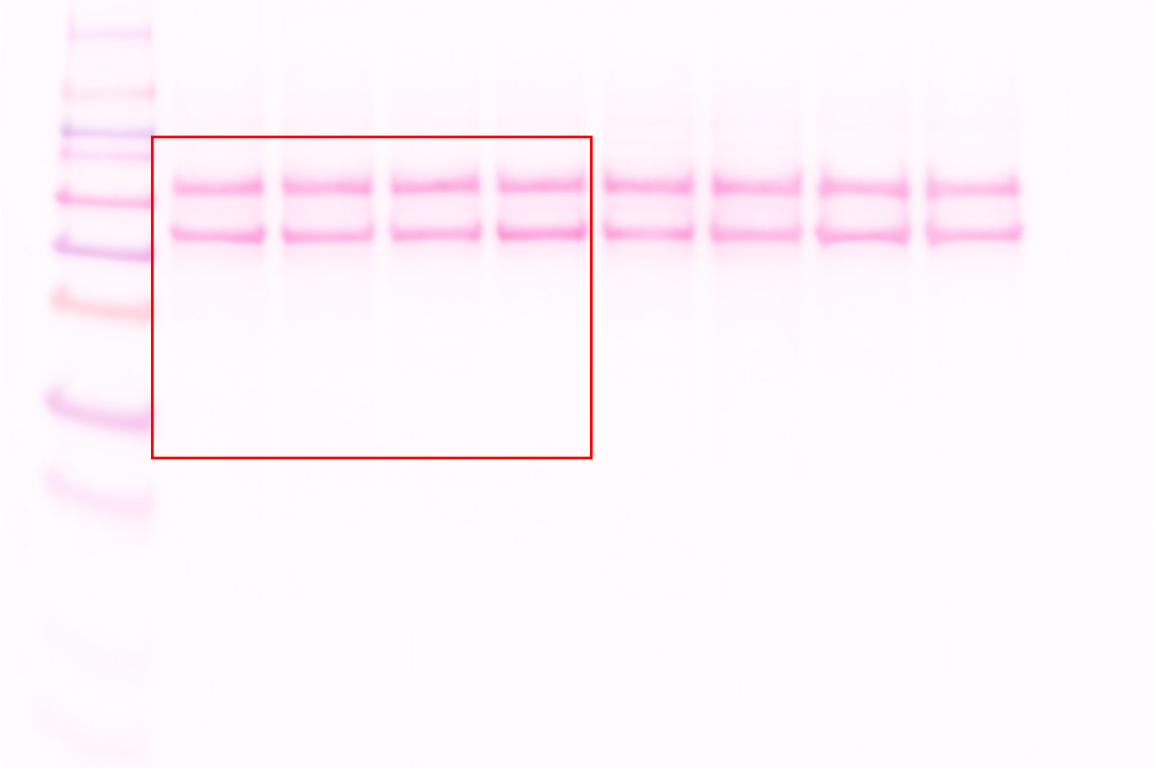

C

IB: Actin

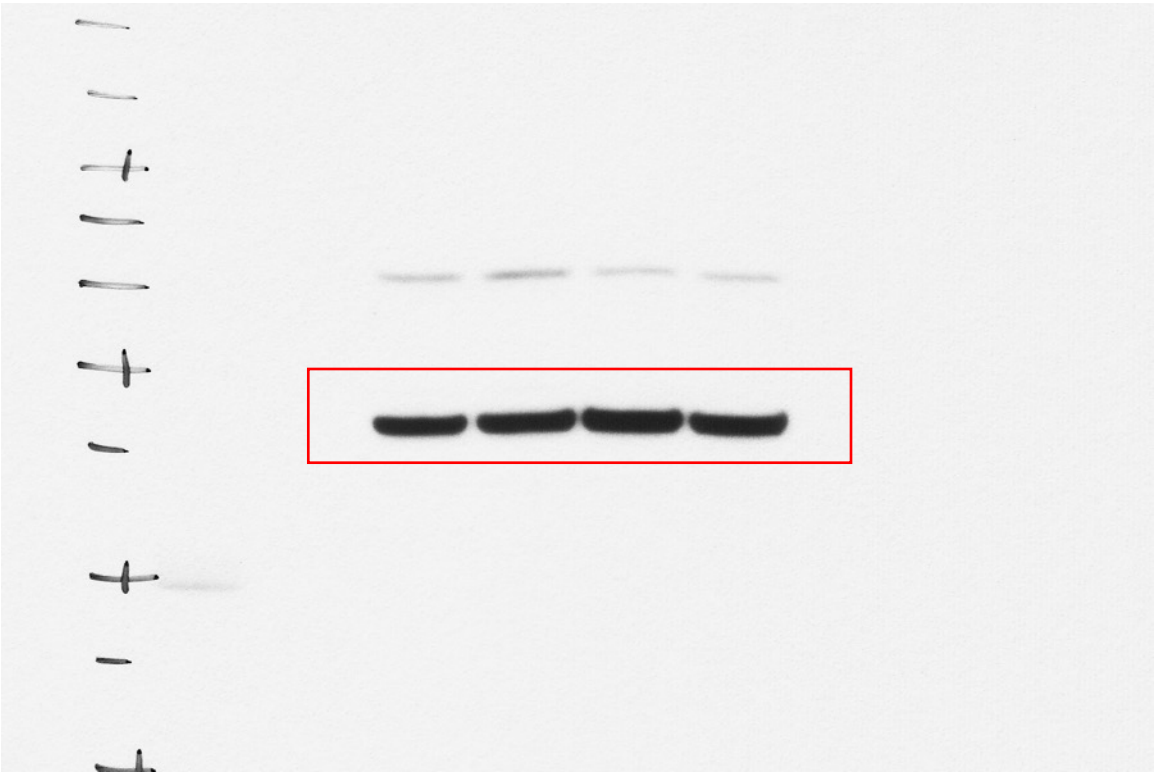

IB:ADAM10

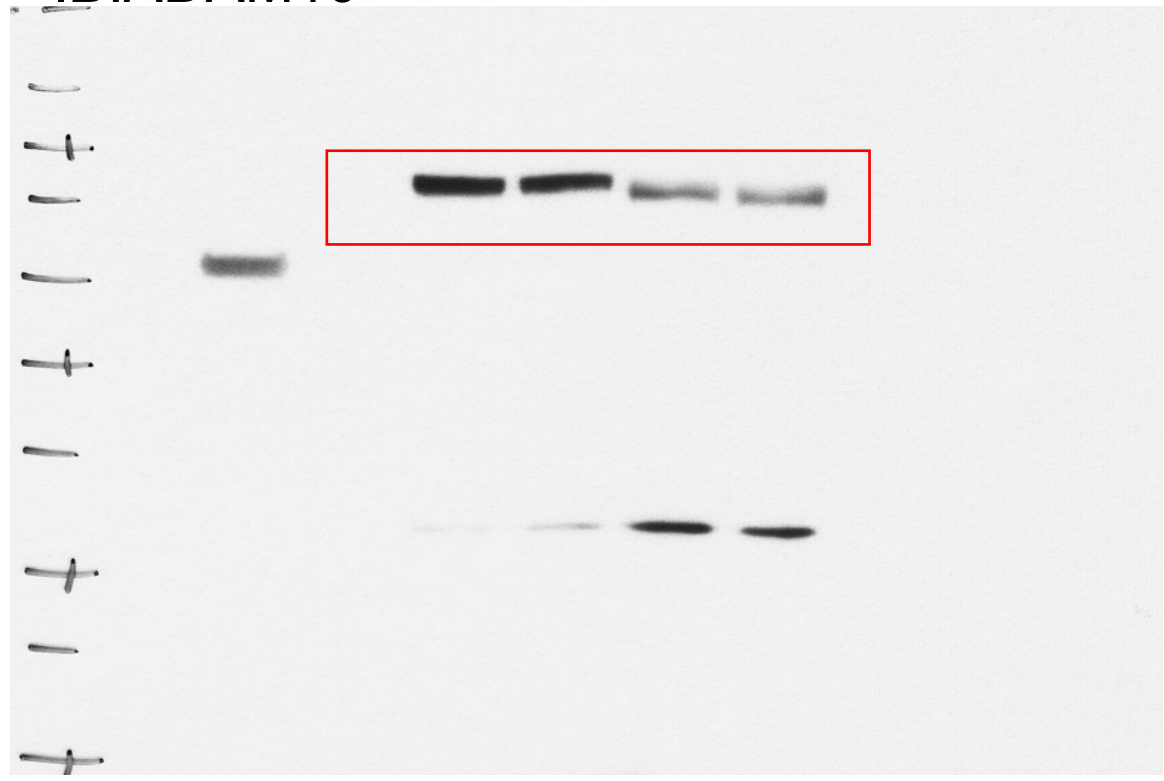

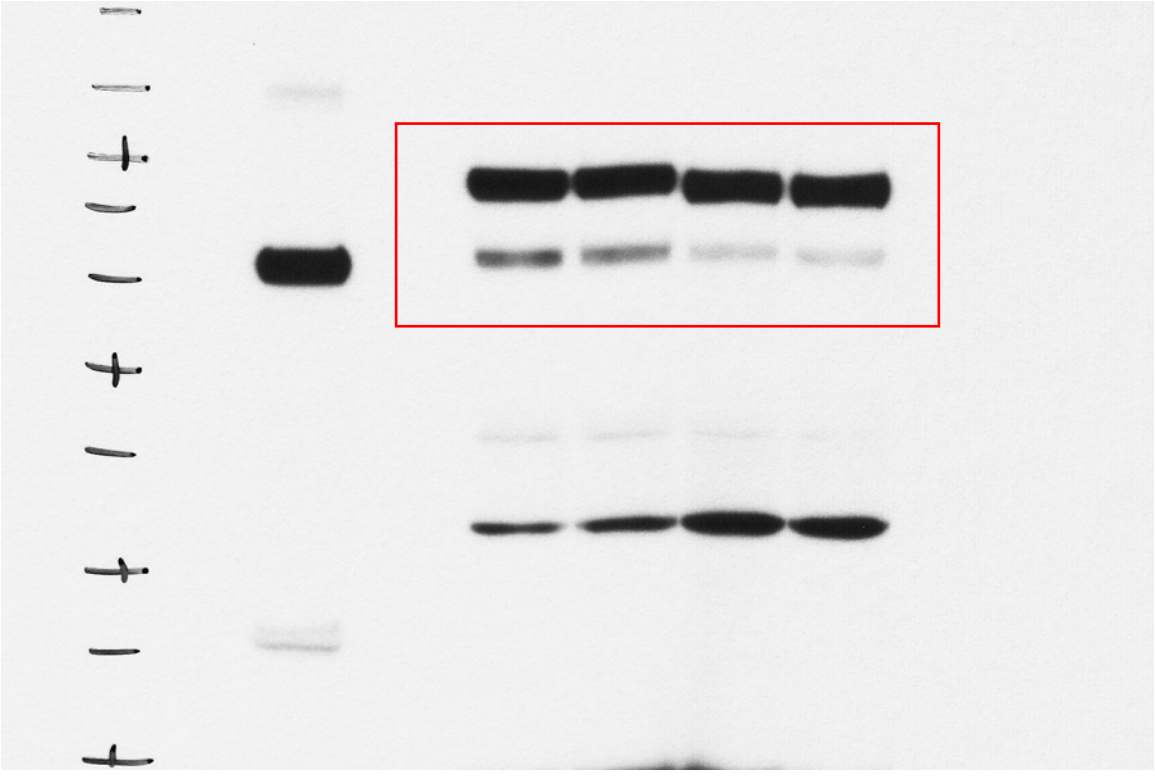

S8 Fig

A (Ctrl, RML)

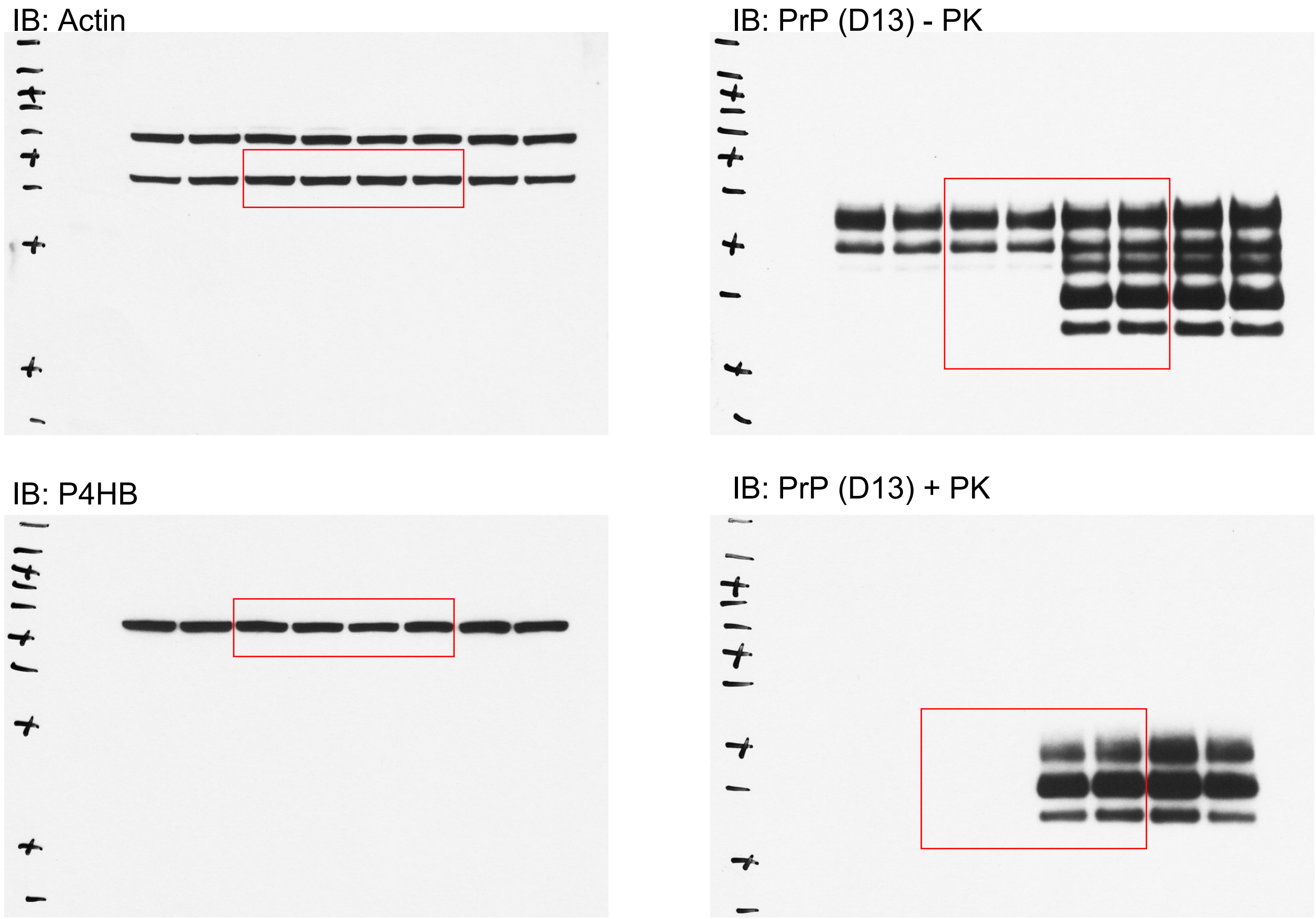

S8 Fig

A (Ctrl, 22L)

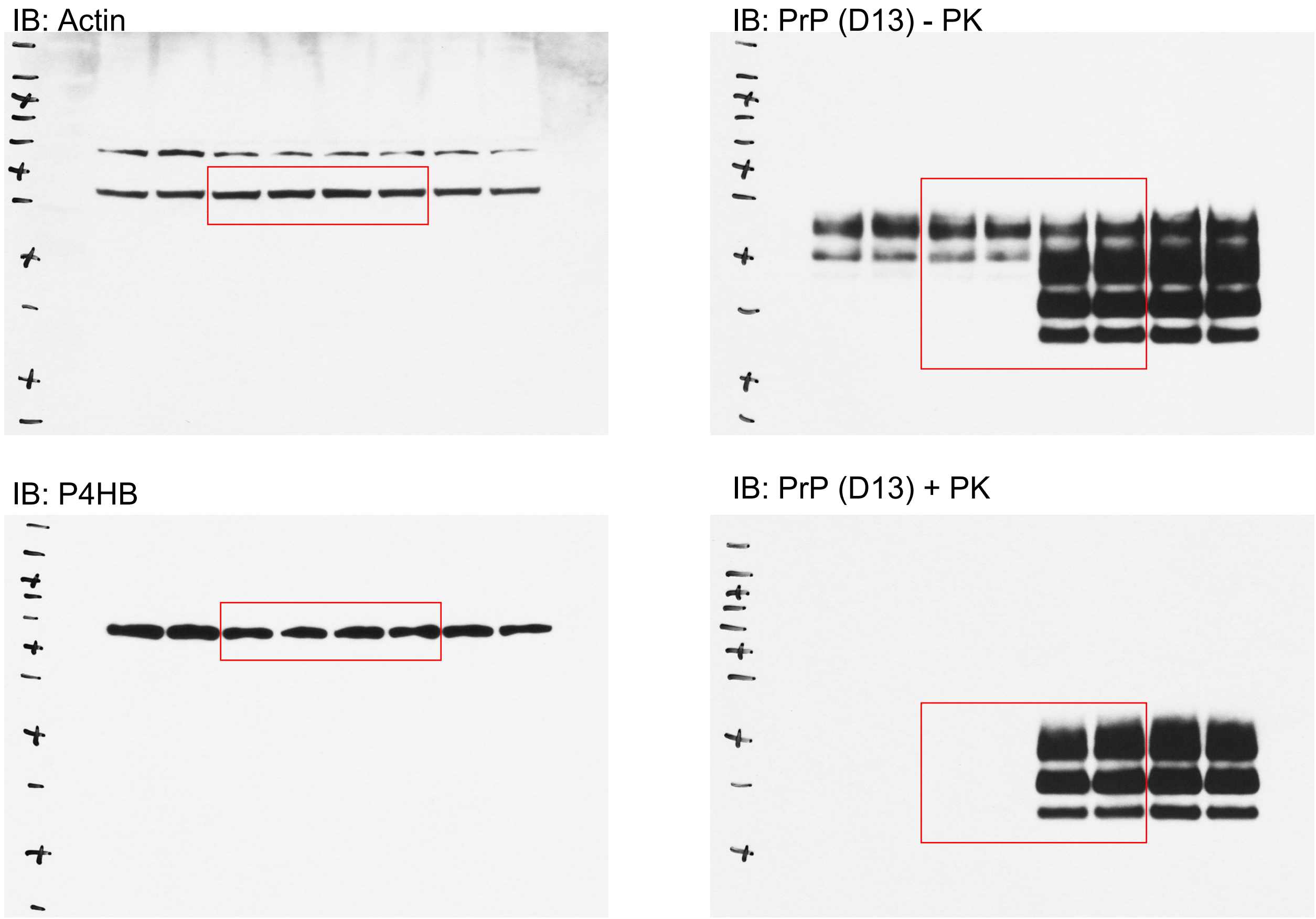

S9 Fig

B

IB: PrP (POM2)

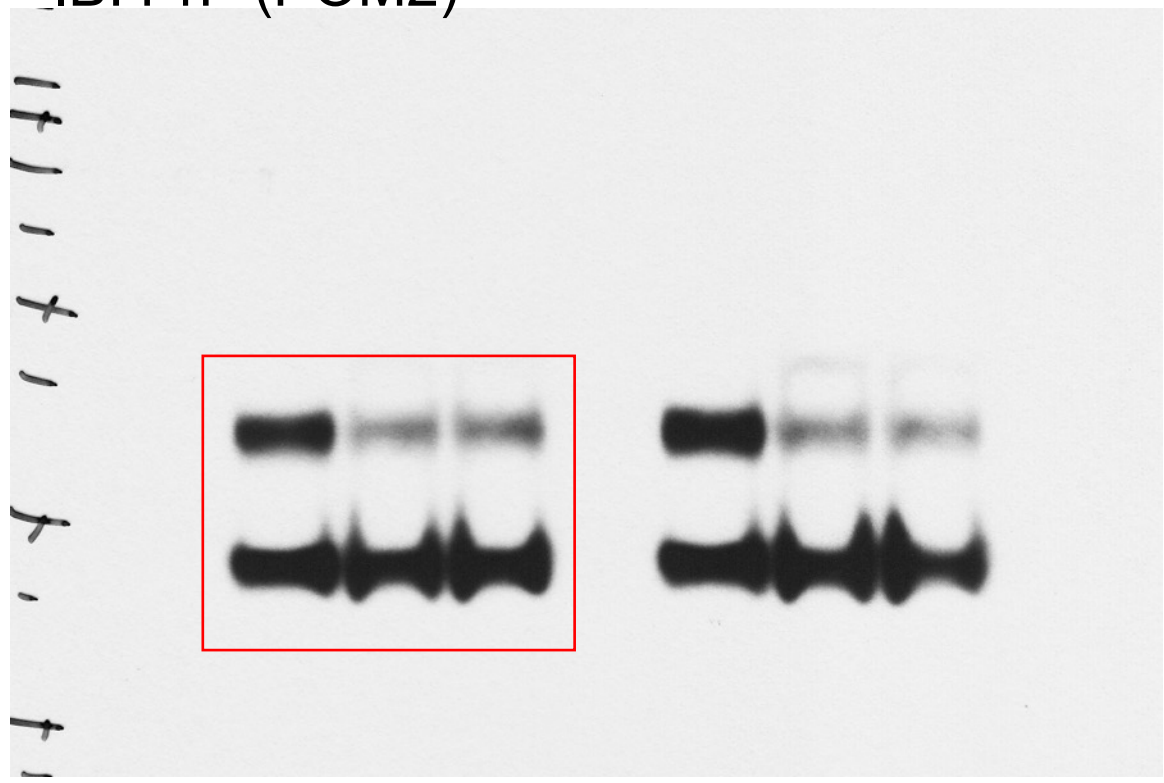

IB: P4HB

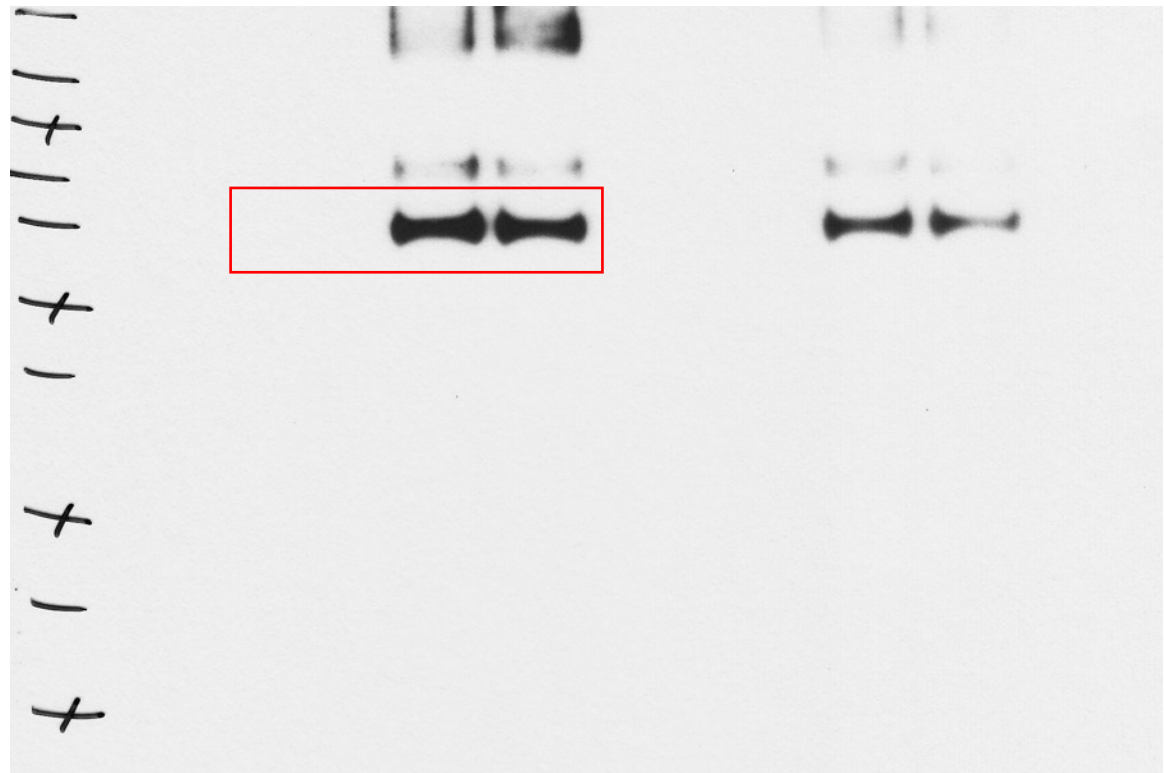

S10 Fig

B

IB: PrP (D13)

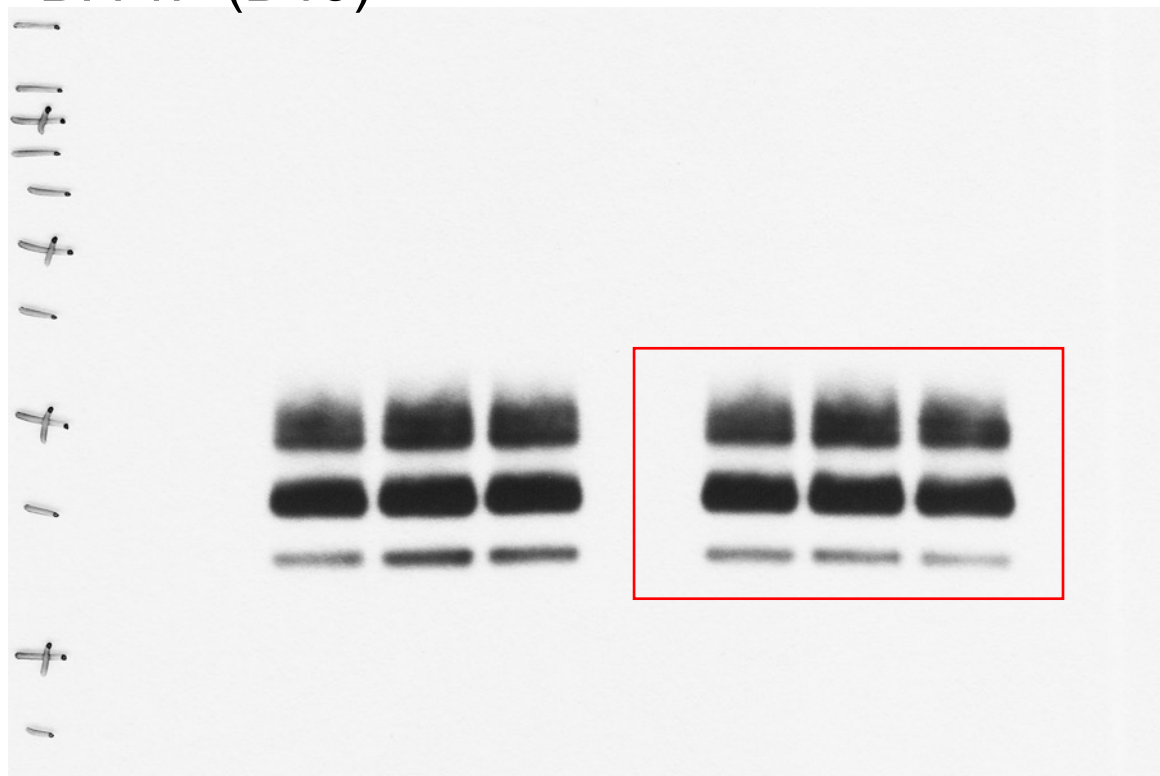

Supplement: Supplementary file 1 [file ppat.1014536.s011.pdf]
